# The Dynamic Energy Band Model of Contact‐Separation and Sliding Mode Triboelectric Charging of Polymers at the Metal‐Polymer Interface

**DOI:** 10.1002/advs.202517094

**Published:** 2026-03-12

**Authors:** Sunay Dilara Ekim, Zelal Yavuz, Tuğba Demir Çalışkan, Hande Güler, H. Tarik Baytekin

**Affiliations:** ^1^ Polymer Science and Technology Program Middle East Technical University Ankara 06800 Turkey; ^2^ UNAM‐National Nanotechnology Research Center Bilkent University Ankara 06800 Turkey; ^3^ Chemical Engineering Department Ankara University Ankara 06800 Turkey; ^4^ Department of Chemistry Middle East Technical University Ankara 06800 Turkey

**Keywords:** polymer crystallinity, material transfer, mechanical stretching, polymer mechanochemistry, triboelectric charging, work function, dynamic energy band

## Abstract

Surfaces are central to human technology, with most historical achievements linked to their manipulation, yet insulating polymers often experience wear, abrasion, and electrostatic charging upon contact with other materials. Understanding the interplay between mechanical deformation and surface charge generation, triboelectrification, is essential for predicting and controlling polymer performance. This study investigates how plastic deformation by strain, a key determinant of polymer mechanical properties, influences triboelectric charge generation. Triboelectrification on stretched and unstretched polymers was measured using two complementary approaches: (i) probing electrical signals at the polymermetal interface during repeated contact separation cycles, and (ii) sliding a metal sphere along polymer surfaces while monitoring interface signals along a linear path. Results demonstrate that structural and physical changes in polymers, including crystallinity and tacticity induced by strain, strongly correlate with charge generation. We propose a dynamic energy band model in which mechanochemical changes at the interface create and decay energy states within the conduction and valence bands relative to the metal Fermi level. This framework accommodates multiple concurrent processes in triboelectric charging. Finally, surface charge density and electron mediated charge transfer can be predicted by evaluating the activation energies of all relevant processes. These findings advance the fundamental understanding of polymer triboelectrification under mechanical deformation and guide the design of materials and devices with controlled surface charging properties.

## Graphical abstract

Triboelectric charge generation at polymer‐metal interfaces arises from the interplay of polymer mechanochemistry, polymer physical properties, and material transfer, where ion‐derived dipoles modulate the interfacial potential and mechanochemically generated electronic states and work function differences drive electron transfer, providing a unified picture of the evolving triboelectric charge density.

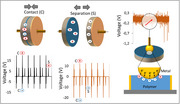

## 1 Introduction

It has been known for a long time that when two surfaces are brought into contact and then separated, or rubbed, friction produces surface electrification, also known as triboelectricity, due to the generation and accumulation of electrical charges on dielectric surfaces.^[^ ^1^, ^2^, ^3^, ^4^, ^5^, ^6^, ^7^, ^8^ ^]^ Triboelectricity occurs from nano to mesoscale when two materials contact or slide against each other. Surface electrostatic charges can contribute to two crucial tribological phenomena, friction and wear, that cause shortening of the lifetime of moving parts in machines and equipment and increase energy and maintenance costs. Some studies have demonstrated that removing triboelectric charges from common polymer surfaces reduces friction and wear, and can decrease power losses in devices with polymer parts.^[^ ^9^ ^]^ Therefore, controlling and reducing tribological effects such as triboelectric charge generation, friction, and wear is a crucial issue due to economic and environmental factors. Accumulating electrical charges on the surface can result in electrostatic discharge (ESD) problems, which can cause serious accidents in the industry or permanently damage electronic devices.^[^ ^10^ ^]^ Conversely, accumulating charges on polymer surfaces is useful in current and future technologies such as photocopying, self‐powered lubricant, biochemical sensors, electrocatalysis, and separation technologies for waste polymers.^[^ ^11^, ^12^, ^13^ ^]^ Triboelectric charging can be observed between different surfaces and interfaces such as insulator‐insulator, metal‐insulator, metal‐metal, gas‐solid, and many different surfaces modified common polymers, and polymer composites have caught the attention of engineers and have been used to generate electrical power devices such as triboelectric nanogenerators (TENGs).^[^ ^14^ ^]^ Although there has been a lot of research on this topic to enlighten the relation between triboelectric charge generation and tribological events,^[^ ^15^, ^16^, ^17^, ^18^ ^]^ especially friction between polymer and metal, one also needs to understand the relationship between physical properties of polymers and triboelectric charge generation at different scales. The primary goals of such research can be given as i) to control triboelectric charging to enhance the output power of triboelectric generator devices, and to dissipate the charge accumulation to avoid the harmful effects of static charging, ii) to form a unified mechanism of triboelectric charge generation.^[^ ^19^ ^]^ In some previous studies, a cross‐relation between tribocharges and friction was shown, e.g., a millimeter‐sized gold ball that was slid on poly(methylmethacrylate) (PMMA),^[^ ^20^ ^]^ alumina‐polytetrafluoroethylene (PTFE) sliding contact in the presence of a lubricant,^[^ ^21^ ^]^ and the friction coefficient of tribocharged PTFE at the macro and nanoscales^[^ ^22^ ^]^ were investigated. At even smaller scales, the strong correlation between friction and triboelectrification was demonstrated by measuring friction and triboelectrification simultaneously during sliding contacts.^[^ ^23^ ^]^

Although many triboelectric systems (energy generators, sensors, wearable materials, etc.) using common and modified polymers have been developed, a disagreement persists regarding the precise mechanism of their operation due to discrepancies in the understanding of triboelectricity. These studies have shown that charging in polymers involves multiple mechanisms, including transfer of electrons from the cloud of electrons on the atoms and molecules,^[^ ^5^ ^]^ ion transfer,^[^ ^6^ ^]^ transfer of charged material,^[^ ^7^ ^]^ and the observation of covalent bond breaking or bond formation due to mechanical forces at the polymer‐metal interface.^[^ ^24^, ^25^, ^26^, ^27^, ^28^ ^]^ These possible mechanisms or related events that can co‐occur, and each mechanism can be influenced by environmental factors differently, make the whole and “’the true”’ mechanism of triboelectricity even more complex.^[^ ^29^, ^30^, ^31^, ^32^, ^33^, ^34^, ^35^, ^36^, ^37^, ^38^, ^39^, ^40^, ^41^, ^42^ ^]^ Surface chemistry, physical characteristics of the polymers (conductivity, roughness, hydrophilicity/hydrophobicity, etc.), chemical (electronical structure, acidity/basicity), mechanical characteristics (hardness, elastic modulus and toughness), and environmental factors (atmosphere, humidity, and temperature) all have a significant role in these mechanisms in the presence of the mechanochemical events during the generation of contact charging. During contact between two rough surfaces, or sliding motion between surfaces, a large strain can stretch polymer chains, dissolve crystallites, extend folded polymer chains, and ultimately rupture covalent bonds. Semicrystalline polymers show physical changes in their structures, like lamellae fragmentation, orientation of fragmented crystal blocks, and new fibril formation due to stretching. Therefore, their mechanical and electrical properties are highly different than unstretched ones. In some studies, material strain^[^ ^37^, ^38^, ^39^, ^40^ ^]^ and heat treatment that changes crystallinity in template‐assisted melt‐pressing^[^ ^42^ ^]^ are related to contact charging, to our knowledge, triboelectric charge generation, and the changes in chemical structure of polymers due to mechanical deformations, considering electronic properties have not yet been reported experimentally in detail.

Here, we demonstrated that mechanical treatment can be used as a strategy to control the tribocharging of an insulating polymer. We initiated our study with semicrystalline poly(propylene) (PP) and poly(tetrafluoroethylene) (PTFE). Some other amorphous and semicrystalline polymers were also employed. These polymers showed either an increase, a decrease, or no change in charging upon uniaxial stretching. PP is widely used in the textile, electronic, and packaging industries; therefore, it is frequently subjected to mechanical deformation in these applications. We first demonstrated that control of triboelectric charging is highly dependent on the changes in mechanical properties. We revealed the structural properties, such as tacticity and degree of crystallinity, as well as the electronic properties that alter the work function of polymers. First, we surmise that new mechanochemical surface states are dynamically created on the polymer surface during physical contact with a metal. Next, and most importantly, we propose that all the proposed mechanisms and theories of contact‐separation and sliding tribocharging can be revealed and unified by considering the band energies that change during contact‐separation and sliding events at the interface. Sliding mode operation is a dynamic process involving mechanical, chemical, and electrical events. During sliding mode operation, strain‐induced and shear forces generate surface mechanochemical states that act as electron‐trapping sites or electron sources within the dielectric material's band gap. Various relaxation mechanisms and changes on the charged surface, including thermal, topographical, and electrical fields, can influence the formation and disappearance of surface states. Surface defects that are formed mechanochemically during the friction^[^ ^32^ ^]^ are continuously generated and lost due to formation, inter,^[^ ^31^ ^]^ and intra^[^ ^43^ ^]^ transformations of mechanochemically produced anions, cations, and radicals. Finally, we hypothesize a dynamic energy band model for the triboelectric charging of polymers, where new energy states are created and decay between the conduction band (E_c)_ and the valence band (E_v_) relative to the position of the Fermi level of the metal as continuous mechanochemical changes occur during the physical contact between the metal and the polymer. Considering the KPFM surface potential measurements and UPS spectra, which are directly related to the work function of polymers, we attribute the changes in contact‐separation and sliding‐mode triboelectric signals to increases and decreases in the work functions of polymers upon stretching and mechanical deformation of the surface. Based on all the spectroscopic evidence and macroscopic modifications, we propose a modified electron transfer model that incorporates the movable (dynamic) energy band of dielectric polymers for contact‐separation and sliding mode triboelectric charge generation. In this model, we assume that the energy band of the metal remains substantially unchanged. Nevertheless, metal surfaces can be modified due to material transfer,^[^ ^7^, ^8^, ^17^, ^44^, ^45^, ^46^ ^]^ which can alter the electronic interactions at the interface, leading to both static and dynamic electrification.

We anticipate that this research will establish a dynamic band energy framework for electron transfer emerging from mechanochemically generated surface states at polymer‐metal interfaces. This perspective may finally illuminate the actual mechanism of triboelectrification, alleviate long‐standing challenges associated with static charging, enable predictive control over material charging behavior, and ultimately elevate the aspects of triboelectric phenomena.

## 2 Results and Discussion

**Figure 1:**
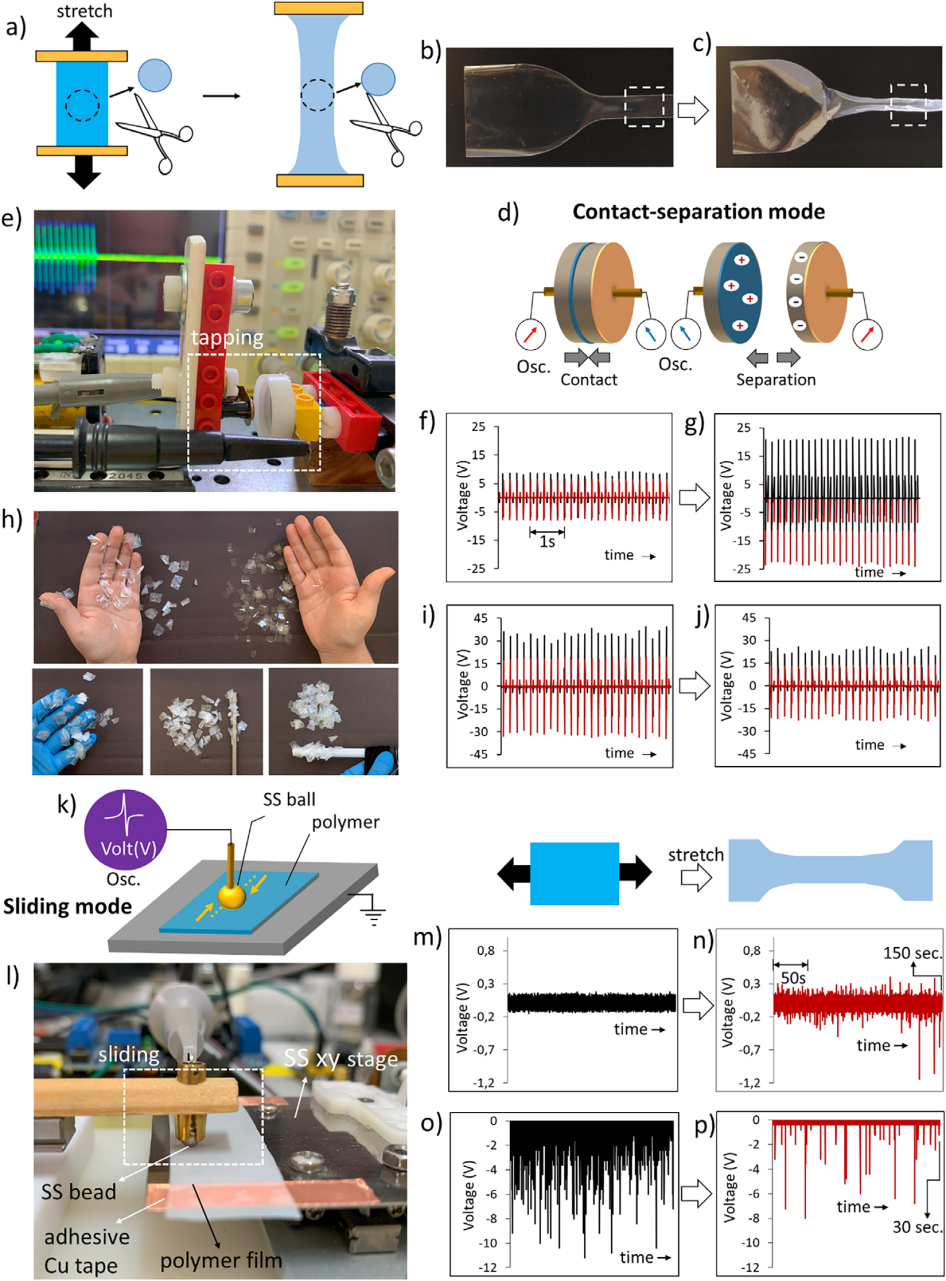
Effects of mechanical treatment on contact‐separation and sliding mode triboelectrification of PP and PTFE films. a) Schematic representation of the preparation of unstretched and stretched polymer films. Photographs of: b) unstretched, and c) stretched PP films. d) Triboelectric charging of contact‐separation electrification. e) Photograph of tapping device. Using an oscilloscope, contact charging electrical signals (VOC) collected from i) the metal electrode positioned underneath the polymer film surface and ii) from a 5‐mm diameter copper disc metal electrode that is tapped to the surface of polymer films at 5.0 Hz frequency. f) Electrostatic charging demonstrating accumulation of stretched and unstretched cut pieces of PP on bare hand skin, on nitrile gloves, on a SS metal rod, and on PTFE rod that was tribocharged by rubbing the rod with velvet and contacted to stretched and unstretched PP pieces. An anti‐electrostatic gun (Zerostat) was used to discharge the (1 cm x 1 cm) PP polymer pieces prior to the contact charging experiments. g) and h) comparison of contact charging of untreated (b) and stretched (c) PP films, and comparison of contact charging of untreated (i) and stretched (j) PTFE films. k) Schematic representation of the setup for sliding triboelectrification. l) Using an oscilloscope, sliding triboelectrification signals are collected from the metal bead that is connected directly to the probe. m) to n) comparison of sliding tribocharging of untreated (m) and stretched (n) PP films, and comparison of sliding tribocharging of untreated o) and stretched p) PTFE films.

We stretched some common polymer films to reveal the effect of mechanical treatment on tribocharging. Untreated (unstretched) and stretched samples were prepared and used in triboelectrification experiments (**Figure** **1**). The applied strain (ε) on the polypropylene (PP) film, with a thickness of 50 µm, was determined during the stretching process using the relation (L − L_0_)/L_0_, where L and L_0_ represent the stretched and initial lengths, respectively. Two different modes of operation (contact‐separation and sliding) were employed using two setups to investigate triboelectric charging developed on plastically deformed and untreated polymer films. The first charging setup enables two‐electrode contact‐separation measurements, where unstretched and stretched polymer films (Figure 1a–c) are placed onto an electrode that is tapped against a copper stub (Movie S4, Supporting Information). The contact charging signal, measured as the open‐circuit potential (V_OC_), was collected from electrodes directly connected to the 100 mΩ probes and recorded using an oscilloscope (Figure 1d,e). Poly(propylene) is one of the most widely used plastics, possessing numerous desirable properties, including flexibility, durability, chemical inertness, strength, lightness, stability, moisture resistance, chemical resistance, and ease of processability, recycling, and reuse.^[^ ^17^, ^47^ ^]^ The effect of plastic deformation on the triboelectric charging of PP was demonstrated using (0.8 cm x 0.8 cm) cut pieces of stretched and unstretched PP; bare hand skin, nitrile gloves, a SS metal rod, and a PTFE rod. After rolling the tribocharged rod over stretched and unstretched pieces of PP, we observed that a higher number of pieces of stretched PP accumulated on the PTFE and SS rods, nitrile gloves, and bare hand skin (see Figure 1h; Movies S1–S3 in the Supporting Information).

Contact charging was developed through repeated contact and separation cycles of two materials, and the resulting data was then collected. The results of contact charging experiments showed that plastically deformed PP film (600% strain) increased contact charging by more than 2.5 times that of the untreated samples, as shown in Figure 1f,g. PTFE exhibited a 30% decrease in contact‐separation electrical signals upon mechanical stretching, as shown in Figure 1i,j. Previous studies have reported that the reversal of charge transfer during contact‐separation charging depends on the radicalic splitting of water molecules, and a change in the work function due to surface deformations.^[^ ^48^, ^49^ ^]^ We observed polarity reversal in the triboelectric contact and separation signal for some of the stretched and unstretched PP films as the number of taps increases (Figure S3, Supporting Information). We attribute polarity reversal to the charge‐stabilization effect of water molecules on mechanochemically generated species, which can be either positively or negatively charged, at the surface.

In the second setup, the SS metal bearing bead slid on the polymer films (Figure 1k; Movie S5, Supporting Information). The bead is placed in a brass metal holder, and PP and PTFE films are directly placed on a 3 cm x 3 cm flat stainless steel (SS) metal plate, which is electrically grounded. The metal bead was slid reciprocating repeatedly on the polymer surface, making a linear motion of 16 mm along the same path. The electrical potential (V_OC_) measured at different times and time intervals is recorded as the metal beads slide on the polymer surface. In this single‐electrode measurement system, the bead is connected directly to the oscilloscope probe using the bead holder. (see Figure S1d, Supporting Information for details of the sliding triboelectrification setup).

The effects of uniaxial stretching on sliding triboelectrification of PP and PTFE films showed two major effects; i) depending on the structure of the polymer, the mechanical treatment has a large effect on the amount of triboelectric charging, and ii) triboelectrification shows a time dependence that indicates both chemical and physicochemical changes that occur on both stretched and unstretched polymers. Rubbing the metal bead on both stretched and unstretched PP generated both positive and negative tribocharging, indicating bidirectional charge transfer (electron flow), as shown in Figure 1n and Figure S2 (Supporting Information). However, unidirectional charge (electron) transfer from the metal to the polymer was observed for both stretched and unstretched PTFE, as shown in Figure 1o,p.

The negative triboelectric signals decreased for both unstretched and stretched PTFE films for 300 s of sliding (Figure S2, Supporting Information). The decrease in tribocharging with increased sliding time for both unstretched and stretched PTFE polymer films suggests that the charge transfer between the metal bead and the polymers is affected by physicochemical changes at the interface. Therefore, we focused on the bead surface and observed that the bead surface at the contact area is partially covered with polymer film due to material transfer, shown in Figure S4 (Supporting Information). Fluorine elemental signals were detected on the bead surface by EDAX analysis after sliding the bead on either stretched or unstretched PTFE films. The material transfer from the stretched film was higher than that from the unstretched PTFE film, as seen in Figure S4 (Supporting Information). The difference presumably occurs due to strain‐induced micromechanisms, such as the alignment/disalignment of polymer chains, recrystallization/melting processes, and the formation/disappearance of fibrillary crystals. Mechanochemical events and transformations involving covalent bond rupture in the polymer result in material transfer from the polymer to the SS metal bead, which can affect electron transfer from the polymer to the counter metal surface. Therefore, a PTFE‐PTFE (bead) identical polymer contact at the interface reduces friction and wear.^[^ ^17^ ^]^ It affects all other related physicochemical and mechanochemical events, as well as electronic properties such as the distribution of surface states and water adsorption (Figure S3, Supporting Information), which directly influence the electronic properties of the interface.

Our experiments using untreated PP and PTFE indicate that triboelectric charging is a time‐dependent phenomenon. Sliding the SS metal bead on the unstretched PP film did not show any significant increase in triboelectric charging up to 1200 s (Figure 1m; Figure S2a, Supporting Information). However, compared to the unstretched PP film, the stretched PP film generated a significantly higher amount of triboelectric charging in a much shorter time, producing randomly generated sharp bipolar electrical signals, as shown in Figure 1n and Figure S2b, Supporting Information. Deformations at the interface, such as wear and abrasive deformation, are the origins of such sharp signals attributed to the sudden dissipation of electrical charges. Triboelectric charging generated from the stretched PP film decreased after 600 s of sliding time (Figure S2b, Supporting Information). Unlike the unstretched PP film surface, we observed visually severe worn and mechanically deformed areas on the stretched film after 600 s of sliding time. The real contact area is reduced due to worn areas appearing along the sliding contact path on the stretched film surface. SEM and AFM surface imaging indicated that the PP film surface is already highly deformed before the sliding due to stretching.

To gain a deeper understanding of the tribocharging effect on stretching polymer films, we investigated the structural changes in the thick films using microscopic and spectroscopic techniques. Polarized microscope image indicates production of cavities^[^ ^50^, ^51^ ^]^ elongated and aligned bright spots on the stretched PP film surface upon uniaxial stretching, Figure S5 (Supporting Information). The chain‐folded lamellar crystal structure of poly(propylene) showed a shear‐induced melting without recrystallization into the chain‐extended fibrillar crystal at 25 °C.^[^ ^49^ ^]^ Therefore, stretching caused a decrease in the crystallinity of PP. Scanning electron microscopy (SEM) and atomic force microscopy (AFM) images of PP and PTFE show the changes in surface morphology and surface deformations upon stretching, as shown in Figures S9 and S10 (Supporting Information).

**Figure 2:**
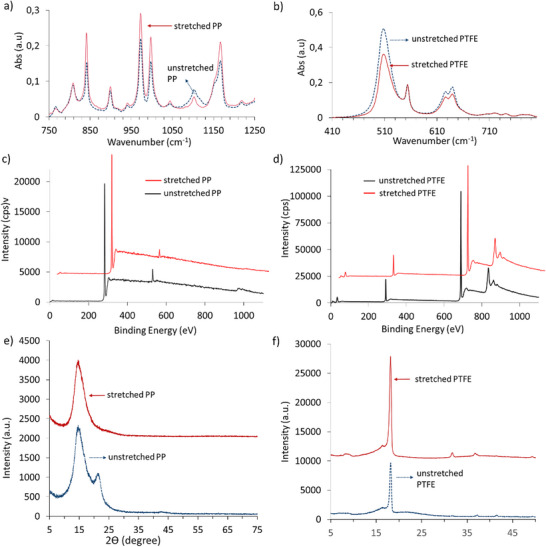
Chemical, structural, and electronic changes upon stretching of PP and PTFE thick films. a) ATR‐IR spectra that are normalized for the symmetric bending vibration peak of CH_3_ group at 1375 cm^−1^ for PP. b) ATR‐IR spectra of unstretched and stretched PTFE. c) and d) XPS spectra of unstretched and stretched PP and PTFE films. e) and f) XRD diffractograms of unstretched and stretched PP and PTFE films.

ATR‐IR peaks are examined to understand the effect of stretching on the structural changes, such as stereoregularity, for PP films^[^ ^52^, ^53^ ^]^ (**Figure** **2**a,b; Figure S5, Supporting Information). We observed an increase in IR absorption at 898 cm^−1^ (─CH_3_ rocking), 974 and 997 cm^−1^ (─CH_3_ rocking, stretching), and an increase at 1168 cm^−1^ due to wagging (C─H), and rocking (─CH_3_) vibrations^[^ ^54^ ^]^ upon stretching the PP film. The ratio of absorption bands at 997 and 974 cm^−1^ was examined to understand the changes in isotacticity.^[^ ^55^, ^56^ ^]^ The ratio ((A_997_/A_974_)x100%) changes from 73.15% to 63.65% when the polymer is stretched. Normalized spectral data at 1375 cm^−1^ (symmetric bending vibration mode of ─CH_3_ group) are present for both unstretched and stretched PP. The absorption bands at 998 and 1168 cm^−1^ increased after stretching. These bands are known to originate from isotactic helices. The helical configuration and long‐range lateral packing influence the intensity of the 998 cm^−1^ band. XPS spectra of unstretched and stretched PP films indicate oxygen contamination on the PP films (Figure 2c). The XRD diffractograms (Figure 2e) show structural changes that are evident from a decrease in crystallinity of the PP film from 60% to 54% upon stretching.

Polymer films were analyzed using the same techniques employed for polypropylene (PP) to reveal the effect of uniaxial stretching on the triboelectric charging of polytetrafluoroethylene (PTFE). ATR‐IR indicates structural changes in PTFE upon stretching.^[^ ^57^ ^]^ Relative intensities of IR signals and peak maxima shifted after the PTFE film was stretched (Figure 2b; Figure S5j,k, Supporting Information). Polymeric bundles were observed on the PTFE film surface along the stretching direction upon stretching using SEM and AFM, Figures S5l–n and S9i–o (Supporting Information). XPS spectra of untreated and stretched PTFE films indicate that no change in elemental composition occurs, Figure 2d. XRD analyses showed that the crystallinity of PTFE film increased from 34% to 43% due to the shear‐driven deformation of slip planes. Structural changes are evident from the significant increase in the crystallinity of PTFE XRD reflection peak that appeared at 2*θ* = 18.2 degree, a significant loss of signal at 36.9 degree, appearance of the peak at 41.62 degree corresponding to the lattice planes (100), (107), and (108), respectively, upon uniaxial stretching^[^ ^57^ ^]^ (Figure 2f). We also analyzed the contacting surface of the SS metal bead by using SEM. We revealed that material transfer occurred from untreated and stretched PP and PTFE films to the metal surface (Figure S4, (Supporting Information)). Changes in physical properties were shown using differential scanning calorimetry. An increase in the melting temperature from 161.5° to 163.8° for PP, and a decrease from 331.9° to 327.7° for PTFE are attributed to strain‐induced changes in the polymer structure (Table S1, Supporting Information). Strain‐induced hardening was observed for PP after stretching using the nanoindentation technique, which is attributed primarily to a strain‐induced increase in the number of elastically active chains. PP showed a significant increase in hardness from 29.4 to 2305 MPa upon 600% stretching using the Rockwell indenter tip. However, the hardness of PTFE decreased from 47.86 to 21.04 MPa upon 70% stretching (Table S2, Supporting Information).

A completely uncharged polymer exhibits a gradual increase in triboelectric signals with repeated contact‐separation cycles. Based on this triboelectrification behavior, we propose generating an increasing number of surface states through mechanochemical reactions occurring in the polymer structure at each contact‐separation cycle. According to the dynamic energy band model, new surface states are continuously generated and decay due to mechanochemical reactions at the polymer‐metal interface. Each of the surface states has an electronic energy assigned to mechanochemically generated groups. R refers to heterolytically and homolytically cleaved polymer chains, or the charged and uncharged moieties on the polymer chain backbone. As the contact number increases, new surface states are generated due to mechanochemical reactions and their density changes dynamically. These “primary” charged groups are currently not fully identified, as their reactivity and stability depend on environmental conditions, such as temperature, humidity, and atmosphere, which are known to significantly impact triboelectrification.

**Figure 3:**
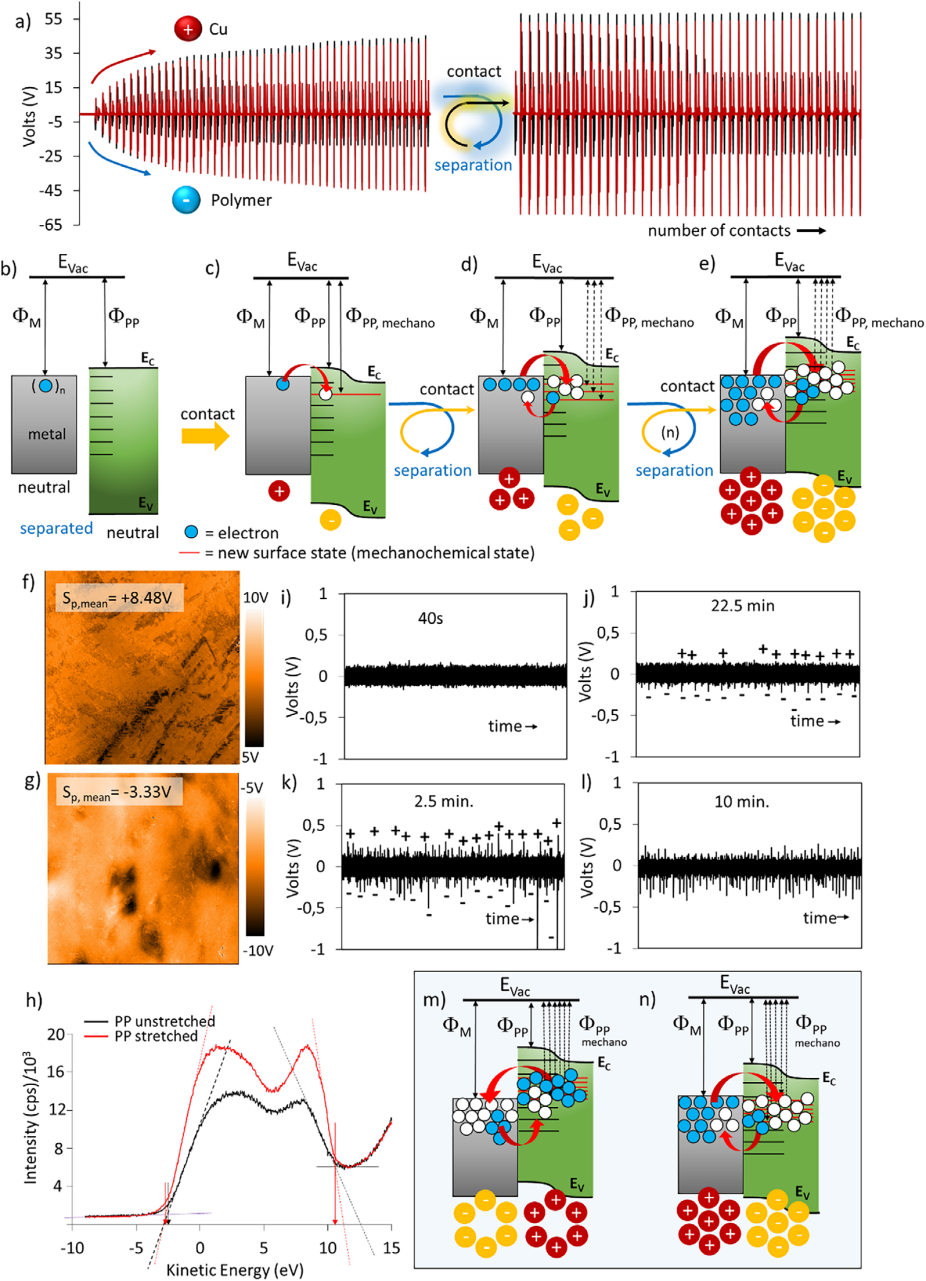
Dynamic band energy model of electron transfer due to interfacial mechanoactivated surfaces states in contact‐separation and sliding mode triboelectric charging of metal‐PP film. a) Contact‐separation triboelectrification of PP film that is tapped using a Cu metal electrode. Contact and separation signals increase for tenths of tapping (left) and reaches to a steady value (right). b) to e) The proposed mechanism of contact‐separation tribocharging: energy band diagrams show the formation of increasing number of new mechanochemical surface states after successive contact‐separation cycles. b) metal electrode and polymer are physically separated and they are neutral before tapping, c) even on the first contact, impact generates mechanochemically created new surface states and electron transfer occurs from metal to machanochemical surface states, d) increased number of tapping generates more surface states and bidirectional electron transfer becomes possible, In (d) and (e) backflow of charge from polymer to metal occurs due to electron transfer from machanochemical surface states. f) KPFM surface potential image showing positive surface potential on unstretched PP film. g) KPFM surface potential image showing negative surface potential of stretched PP film. h) UPS spectra of unstretched and stretched PP films. i) and j) sliding triboelectrification of unstretched PP film obtained at two different times. k) and l) sliding triboelectrification of stretched PP film obtained at two different times. m) and n) energy band diagrams showing new mechanochemical surface states (red lines) and the formation of positive and negative triboelectric signals due to bidirectional electron transfer in sliding mode triboelectrification.

The accumulation of charges appearing as an increase in triboelectric signals and saturation of signals in contact‐separation charging of PP are given in **Figure** **3**a. The entire energy band of the polymer can extend to higher energy levels, as illustrated in Figure 3b–e for PP. UV‐Photoelectron spectroscopy (UPS) measurements reveal that the electronic energy levels of the bands change upon strain. According to the literature, untreated PP has a higher work function (W_f_ = 5.49 eV)^[^ ^58^ ^]^ than that of SS metal (W_f_ = 4.08–4.19 eV),^[^ ^59^ ^]^ and Cu (4.35 eV),^[^ ^60^ ^]^ and that of Cu_2_O ≈(4.89 eV).^[^ ^61^, ^62^ ^]^ Therefore, charge transfer is due to the electron flow from the lower work function metal to the PP polymer. In this case, the polymer becomes negatively charged. Determination of the work function of insulators or molecules using UPS requires conductive samples or thin‐layer samples for insulators on standardized pure metal substrates. Due to practical reasons, we decided to compare the relative changes in work functions of mechanically treated and untreated polymer thick samples, which were measured under the same conditions. UPS measurements reveal that the work function decreases by 0.22 eV (Figure 3h) upon stretching the PP film, indicating that stretching reduces the energy barrier and facilitates electron transfer to the amorphous PP film. As a result of the stretching of PP, electronic modification occurs, and the triboelectric signals increase in both the contact‐separation mode and the sliding mode of triboelectrification between metal and polymer.

Starting from the first contact, periodic cycles of increase and decrease in the net triboelectric signals might be observed due to the random distribution of surface states after each tapping. Electrostatic air breakdown can be effective, depending on the charge dissipation due to humidity. Both tensile and compressive strains are exerted on the polymer chains in the contact‐separation and sliding modes of triboelectrification, to varying extents. Strain applied to organic molecules can change their work functions.^[^ ^63^ ^]^ We obtained KPFM images that show the surface potential distribution over the unstretched and stretched PP film surfaces. KPFM surface potential maps of unstretched PP samples indicate a charge heterogeneity at different measurement areas, as shown in Figure 3f. However, stretched PP surfaces exhibit negative surface potentials obtained from different areas, as shown in Figure 3g. We observed both positive and negative electrical signals during sliding‐mode triboelectrification of unstretched (Figure 3j) and stretched polypropylene (PP) films (Figure 3k,l). According to the dynamic energy‐band model of sliding triboelectrification, we propose that new surface states are continuously generated as the metal bead slides on the polymer surface. As the sliding time increases across repeated cycles on the same path, the population of surface states increases, leading to a higher number of tri across repeated cycles on the same path, the population of surface states increases, leading to a higher number of triboelectric signals (Figure 3m,n). Due to continuous physical contact along the sliding path, forward and reverse charge transfers occur simultaneously, resulting in bipolar triboelectric signals in either the forward or reverse direction of sliding. Compared to unstretched polypropylene (PP) film, the generation of triboelectric signals appears in a shorter time and at higher intensities for the stretched PP film.

**Figure 4:**
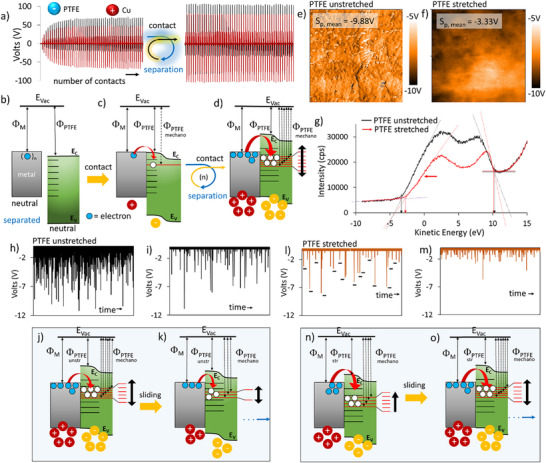
Dynamic band energy model of electron transfer in contact‐separation and sliding mode triboelectric charging of metal‐PTFE film. a) Contact‐separation electrification of initially uncharged PTFE film that is tapped using a Cu metal electrode. Contact and separation signals increase for tenths of tapping (left). b) to d) Energy band diagrams showing the proposed mechanism of contact‐separation tribocharging of PTFE film that is tapped using a metal electrode. Energy band diagrams show the formation of increasing number of mechanochemical surface states (red lines) in repetitive contact‐separation cycles. e) KPFM surface potential image showing negative surface potential of unstretched PTFE film. f) KPFM surface potential image of stretched PTFE film. g) UPS spectra of unstretched and stretched PTFE films. h) and i) sliding triboelectrification of unstretched PTFE film obtained at two different times (Figure S2, Supporting Information). j) and k) Dynamic band energy model of electron transfer in contact‐separation triboelectric charging of PTFE film for unstretched PTFE. l) and m) sliding triboelectrification of stretched PTFE film obtained at two different sliding times (Figure S2, Supporting Information). n) and o) Dynamic band energy model of electron transfer in contact‐separation triboelectric charging of stretched PTFE film. Note that the formation of only negative triboelectric signals due to unidirectional electron transfer from metal to PTFE in sliding mode triboelectrification was observed in all cases for PTFE.

According to DFT calculations, PTFE shows a very large HOMO‐LUMO gap (HOMO: −8.61 eV, LUMO: −1.60 eV), and PP has 9.67 eV (HOMO: −7.43 eV, LUMO: 2.24 eV). Therefore, triboelectric charging cannot be explained by a simple Fermi‐LUMO alignment argument. The energy bands of polymers contain energy states within this gap that act as charge trapping sites. These mid‐gap states enable localized charge stabilization even though the pristine polymer electronic structures cannot directly exchange electrons with the metal's Fermi level. Most metals have work functions of 4.0‐5.5 eV, placing their Fermi levels far from the LUMO and HOMO levels of both PP and PTFE. This energy mismatch impedes direct electron transfer, rendering orbital alignment insufficient to account for the observed charging. Instead, the triboelectric response is governed by defect‐derived trap states and mechanochemically induced transient states that arise under friction, deformation, and local bond rearrangement. These localized and stress‐generated intermediary states constitute the true pathways controlling the tribocharging behavior of PP and PTFE.^[^ ^63^ ^]^ Deformation of the backbone of poly(tetrafluoroethylene) (PTFE) has been theoretically shown to result in more electron‐deficient carbon atoms, thereby increasing the local dipole moment along the chain. The contact force decreases the LUMO level and enhances the electron‐accepting tendency of PTFE.^[^ ^64^ ^]^ From this theoretical study, we can infer that changing the crystal structure of PTFE by a force is expect to alter triboelectric charging due to changes in the energy levels since the energy gap is decreased as the deformation is strengthened. We used a semicrystalline PTFE to investigate the effect of mechanical deformation, specifically stretching, on triboelectric charging behavior. We observed that PTFE is negatively charged due to unidirectional charge transfer from the metal to the polymer, in both contact‐separation and sliding mode triboelectrification. Starting from a completely uncharged surface, the net charge accumulation shows a gradual increase in electrical signals after contact separation cycles (**Figure** **4**a). It has been previously reported that the density of acceptor states that correspond to LUMO energy levels, that is, located around the carbon atom (proposed as dangling species of carbocation C^+^ and carbon atom radical C),^[^ ^26^ ^]^ remains constant under steady‐state contact charging in which electron transfer occurs from metal to PTFE polymer.^[^ ^65^ ^]^

The energy band diagrams illustrate the flow of electrons from the metal to the polymer surface states of PTFE (red lines) during repetitive contact‐separation cycles, as shown in Figure 4a. Most importantly, the dynamic electronic nature of the surface states results in a dynamic energy band diagram due to the changing potential energy of the surface upon repetitive contact and separation cycles. After many contact‐separation cycles, the surface charge density reaches a steady state, as shown in Figure 4a, right.

The net rate of formation of surface states primarily depends on the electronic structure of PTFE and the surrounding environmental conditions during mechanochemical activation of the polymer chain. Therefore, PTFE behaves differently than PP; it charges only negatively, since mainly radicalic entities are generated and stabilized upon mechanochemical activation. An experimental and theoretical study on the mechanism of PTFE film transfer to metal points out that various possible reactions, such as defluorination, carbonyl and hydroxyl substitution on the chain, chain scission, and reaction with H_2_O and O_2_, can take place. Defluorination, which produces a carbon radical atom first, then a reaction with water generates carbonyl groups on the PTFE chain backbone, resulting in the least energy difference between the transition state and the reactant polymer chain. Electron flow from metal to PTFE in all cases due to the higher work function of PTFE (W_f_ = 5.49 eV),^[^ ^66^ ^]^ and therefore PTFE accumulates only negative charge. However, theoretically, it has been shown that pristine PTFE cannot become stable, either when it accepts or loses electrons.^[^ ^32^ ^]^ Therefore, a work function‐based electron transfer mechanism between metal and polymer, without considering the mechanoactivated species at the interface, cannot be used to account for triboelectric charging of polymers without using a mechanochemical approach.

KPFM surface potential maps of unstretched PTFE samples indicate a negative homogeneous charge distribution, as shown in Figure 4e. However, stretched PP surfaces exhibit less negative surface‐average potentials at all, obtained from the different areas, as shown in Figure 4f. UPS measurements revealed that the work function of PTFE increased by 0.73 eV (Figure 4g), indicating that electron transfer and triboelectric charging are diminished as the crystal structure of the PTFE film increases due to stretching. The number of intense triboelectric signals decreases as the sliding time is continued for both stretched and unstretched films. This observation suggests that the population of mechanochemical surface states decreases over time for the unstretched film, as the material transfers from PTFE to the metal bead (Figure S4, Supporting Information), modifying the polymer‐metal interface at the contact and significantly affecting charge transfer. In other words, the surface dipole density changes over time differently for both films, and its effect on the triboelectric signals is evident in Figure 4h,i for unstretched PTFE, and in Figure 4l,m for stretched PTFE, respectively. The dynamic energy band diagrams of unstretched PTFE exhibit a decrease in the number of triboelectrical signals upon increasing the number of repeated sliding motions, as illustrated in Figure 4j,k, resulting from reduced interfacial interactions due to material transfer during sliding motion. According to the dynamic energy band model, a decrease in the number of surface states results in a reduction of the total electrostatic potential energy of the unstretched PTFE surface, causing the band to move downward.

On the other hand, stretched PTFE generates significantly fewer intense triboelectrical signals compared to the unstretched one, as the electron‐accepting property is diminished, compare Figure 4j–n. The number of surface states, and hence, triboelectric charging, increases as the number of contact separation cycles increases, as shown in Figure 4a. Similarly, as the sliding time increases, the number of triboelectric signals increases, as seen in Figure 4l,m. The energy‐band model states that an increase in the number of surface states increases the total electrostatic potential energy of the stretched PTFE surface, thereby shifting the band upward. Nevertheless, the triboelectric charging decreases upon stretching, see and compare Figure 4h,l.

XPS analysis showed that the PTFE surface did not exhibit any oxygenated species after physical contact, indicating that physical contact did not chemically modify the PTFE surface. However, the analysis of the residue on the counterface metal indicates that bond scission takes place, evidenced by XPS as shown in Figure S8 (Supporting Information). The unidirectional transfer of material that alters the work function underscores the significance of the interfacial material composition.

According to the dynamic energy band model of triboelectricity, both the amplitude, sign, polarity reversal (bipolar in PP and unipolar in PTFE), and the number of triboelectric signals depend on the position and density of mechanochemical surface states associated with the interfacial polymer moieties. The mechanism of charge generation of PP and PTFE is initiated with the mechanochemical activation (scission/atom or group transfer) of polymer chains on the surface.^[^ ^32^, ^33^ ^]^ The possible intra‐ and inter‐transformation of these three mechanochemical moieties (R·, R^+^, and R^−^) appears extremely complex due to uncertainties in their exact identities, their abundances, and the rate of their transformation reactions, as well as their electronic interactions with the metal's electrons and vacant sites at experimental conditions. At that point, stabilization of (+) and (−) charges by humidity, yielding charged water clusters, free radical reactions with water^[^ ^36^ ^]^ and O_2_^[^ ^67^, ^68^ ^]^ as well as chemisorption and physisorption of other gases onto the polymer surface, modify the metal‐polymer interface and can alter the electron transport rate due to dynamical changes in the work function of interfacial moieties. Consequently, any physicochemical change that alters the work function affects signal intensity and electron migration behavior across the interfaces in triboelectrification. Depending on the stabilization of mechanochemically charged species, water deposition may shift the energy band, leading to either a decrease in negative charging or an increase in positive charging and potentially a polarity reversal in triboelectric signals.

There are substantial differences between contact and separation modes, as well as between contact‐separation and sliding modes, considering the nature of the physical interaction between the metal and the polymer. Differences in interactions across various modes result in distinct V_OC_ waveforms in the triboelectric signals, as illustrated in Figure 3 for PP and Figure 4 for PTFE. For instance, compression of the softer polymer surface by the more rigid, hard, and rough metal surface yields mechanochemical reactions due to the localized high‐strain and shearing forces on the polymer chains at the nano‐sized asperities. In this mode, intermolecular attractive forces, along with other physical interactions such as the diffusion and filling of polymers into metal voids and chemisorption processes, occur when an external force is applied. As a result of these interactions, polymer chains adhere to the metal, providing conformal contact between the surfaces, and charge generation at the interface in contact mode results in contact triboelectric charging. Upon separation, which is caused by an external force, all these physical interactions must be overcome, and surfaces are physically separated from each other. The charge generation at the interface in separation mode results in separation‐triboelectric charging. If the polymer is tensioned between two surfaces during separation, chain scission occurs, resulting in anionic/cationic or radicalic sites. Chemisorption and some physical interactions cause material transfer to occur even after the first contact‐separation cycle.^[^ ^60^, ^69^ ^]^ Sliding motion triboelectrification between a metal bead and a polymer film can be envisioned as the simultaneous occurrence of contact and separation processes at the interface. Therefore, the triboelectric signals appear very randomly due to the complex nature of dynamic topographical and physicochemical processes, such as wear, abrasion, changes in real contact area resulting from roughness/flattening events, structural changes like crystallinity and tacticity, reactions with water,^[^ ^36^ ^]^ O_2_,^[^ ^68^ ^]^ and uni‐ or bidirectional material transfer at the interface. All these possible processes may result in the random generation of surface states during sliding motion; therefore, sliding triboelectrification is a complex and unpredictable phenomenon.

**Figure 5:**
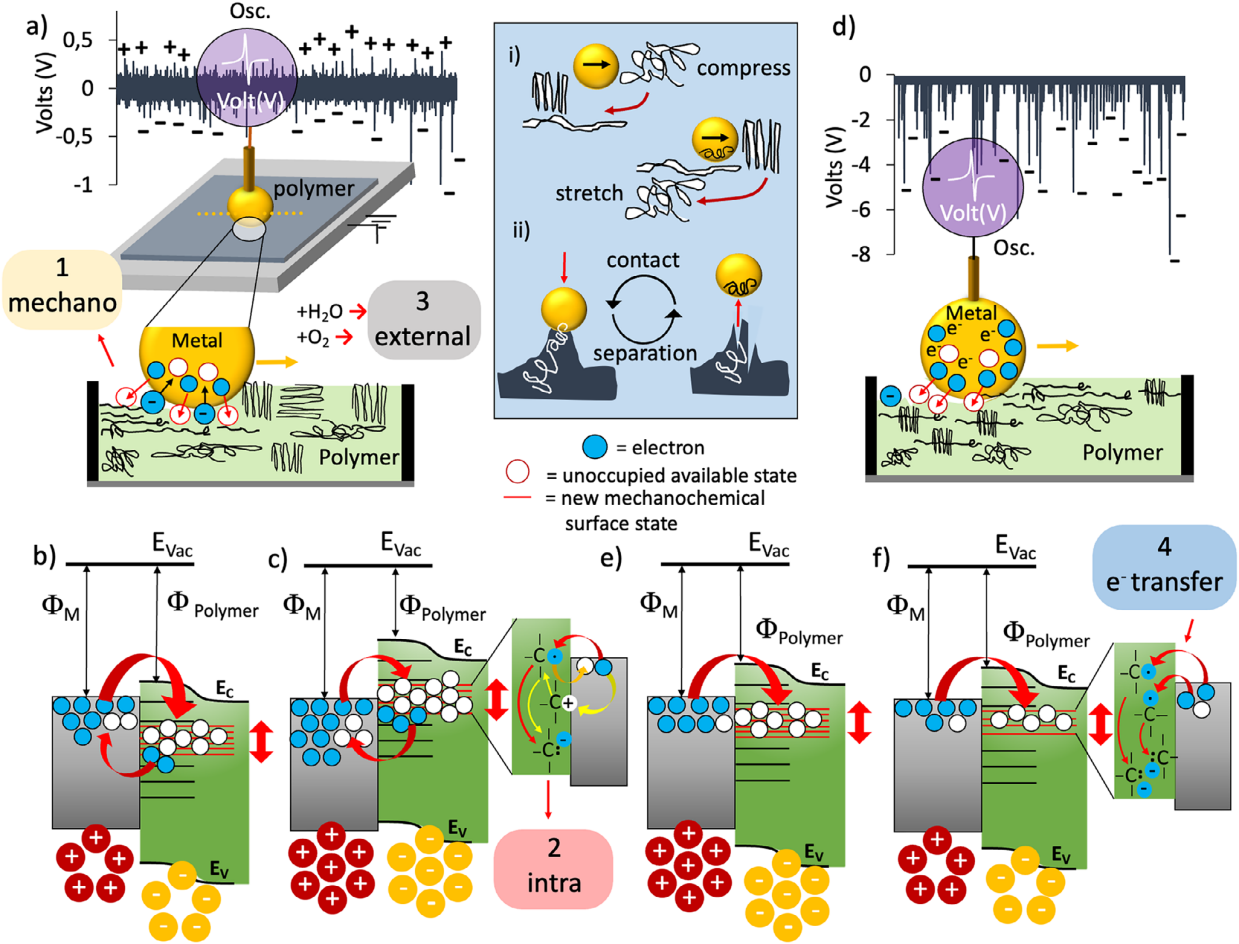
Proposed mechanism of contact‐separation and sliding triboelectrification: mechanoactivation generates new surface states on the polymer surface during the interfacial contact. Dynamic surface mechanochemical states are continuously generated and decayed due to covalent bond rupture either in contact‐separation or in sliding mode. a) sliding mode triboelectrification produces positive and negative triboelectric signals in unstretched or stretched PP. b) and c) shows the dynamic energy band diagrams and bidirectional electron transfer “’from and to”’ the mechanoactivated polymer: mechanoactivation generate positive and negative active sites and charge transfer occurs either by accepting electrons or donating electrons from these active sites. d) sliding triboelectrification of PTFE generate only negative charges due to one‐way electron transfer from metal to PTFE. e) and f) shows the dynamic energy band diagram and unidirectional electron transfer from metal to mechanoactivated surface states of a polymer.

The dynamic energy band model with dynamic energy bands is illustrated in **Figure** **5**. UPS measurements and KPFM surface images revealed that the energy band diagrams of the unstretched and stretched polymers lie at different energy levels. Upon contact‐separation and sliding motion, new surface states whose energies lie between the conduction band (E_c_) and the valence band (E_v_) are generated and lost dynamically. Therefore, the work function and the conduction band edge E_c_ of PP (and PTFE) change dynamically as new mechanochemical surface states are generated (and also decay) at each contact‐separation and during the sliding motion. As the number of mechanochemically generated charged groups changes at the polymer surface (Figure 5b–f), the density of surface dipoles also changes.^[^ ^70^ ^]^ As a result of these events, the work function is modified due to the changing density of surface dipoles,^[^ ^71^, ^72^ ^]^ as described by Helmholtz's equation,^[^ ^70^, ^73^ ^]^ and the triboelectric signal intensity also changes accordingly. The flow of electrons from the metal source to the mechanoactivated charged (R^+^, and R^−^), and uncharged (R) groups,^[^ ^31^ ^]^ or vice versa, is illustrated in Figure 5c–f.

Charge transfer is strongly influenced by material transfer, as the metal's work function is altered by the permanent transfer of polymer residue onto the metal after separation. However, due to the limited surface coverage, the metal's work function is expected not to change significantly. Although the Fermi level is considered fixed, the work function at the metal‐polymer interface depends on the vacuum energy level E_vac,_ which can shift to higher or lower energy levels depending on the orientation of interface dipoles formed by mechanochemically activated polar and radicalic sites.^[^ ^74^, ^75^ ^]^ These electronic modifications at the interface tune the metal's work function and may result in an efficient match with the mechanochemical surface states, causing an increase in tribocharging. In contrast, inefficient matching diminishes the charge injection, causing a decrease in tribocharging.

Surface modification following mechanochemical bond rupture (e.g., oxidation, water deposition, and subsequent reactions, as well as acidic‐basic reactions induced by water) is crucial for understanding the dynamic behavior of mechanochemically generated surface states. It has been demonstrated that the presence of water significantly decreases the wear rates of PTFE due to mechanochemically generated carboxylate end groups, which diminish the transfer of polymer films to the steel substrate.^[^ ^76^ ^]^ Most importantly, mechanochemically formed carboxylic acid end groups and steel are shown to create a chelation in the presence of humid air. Due to chelation or chemisorption, metal‐polymer bonding can introduce a dipole that contributes to the total surface charge density, given by σ = *Q*/*A*. Q is the surface charge, and A is the area on which the charged species exists.

To reveal the charging event in contact‐separation and sliding mode triboelectrification, we divided the whole event into three main parts. We conceived the processes that are involved as follows: first, mechanochemically initiated reactions at the metal‐polymer interface generate (+/−) charged surface groups and radicalic moieties, anywhere at the polymer backbone or at the atoms at the end groups of the ruptured chain. These groups exhibit a dipole moment due to uneven electron distribution, and each moietycorresponds to a dynamic surface state at definite energy. The electron transfer occurs between the mechanochemically formed surface states and the metal. Based on the experimental data, triboelectric charge measurements show charging increases at each contact, and then the triboelectric signals become constant. Second, as a result of the accumulation of net charges at the polymer surface, the electric field affects the electrons of the metal inductively, and “tribo‐induction signals” appear as an additional waveform in addition to “triboelectric” signals. We also hypothesize that the triboelectric potential generated by the induction effect directly depends on the surface charge generated during contact and separation, or on the polymer's charge density at those processes. The induction signal increases along with the triboelectric signals and becomes constant as the number of contacts increases due to the constant (steady state) rate of mechanochemical reactions and all the other processes. For simplicity, we have ignored the induction effects that originate from polarization and concentrated on the surface charge generation and transfer of charges between the metal and the polymer.

Mechanochemistry points out that depending on the chemical structure of the polymer, bond scissions occur heterolytically, homolytically,^[^ ^77^ ^]^ or both at the metal‐polymer interface, resulting in polymeric fragments with (+/−) and radicalic end groups. As mechanochemical reactions occur at the interface, new surface states are generated, and the electrostatic potential energy of the polymer increases due to new surface states. Therefore, the energy band of the polymer that contains mechanochemical surface states shifts higher (or lower) depending on the net dynamical dipole density of the polymer surface. A change in dipole density due to dynamical mechanochemical reactions modifies the work function, as described by the Helmholtz equation given as;^[^ ^70^ ^]^

(1) $$\Delta W_{C,S}=-\frac{e\left(\left[N_{a}\right]+\left[N_{b}\right]+\left[N_{c}\right]+\left[N_{d}\right]\right)}{\varepsilon_{0}}$$

where Δ*W_C_* is the change in work function (the difference between the work function of the pristine polymer and that of the contacted polymer (1st, 2nd, and 3rd. cycles) at the interface in the contact electrification process. The first term represents the dynamic dipole moment density at the interface at the contact, and after the separation, and during sliding motion. The second term is the dipole moment density of the chemisorbed polymer layer on the metal, arising from possible material transfer. Mechanochemically detached polymer fragments transfer onto the metal surface and form a Helmholtztype dipole layer. The resulting dipole layer decreases the number of accessible metal sites for electron exchange, alters the interfacial energy alignment required for electron transfer, and introduces dielectric screening that suppresses radical‐mediated pathways. The previous history of contact, therefore, determines the material's charging behaviors.^[^ ^35^ ^]^ Similarly, Δ*W_S_* is the change in the work function of the polymer after physical separation in the separation electrification process. Here, [*N_a_*], [*N_b_*], [*N_c_*] and [*N_d_*] are the dynamic dipole moment densities of the active sites that bear (+), and (−) charges, and radical moieties, and donor acceptor complexes (D^+^‐ A^−^), respectively, reveals the distribution of charges and dipole‐carrying mechanochemical moieties, on the surface, which directly correlates with changes in the work function and e is the elemental charge. [*N_d_*] is the dipole moment density associated with the polymer's deposition on the metal, which depends on the type of metal and surface lattice, and the environmental conditions. The surface potential measured by KPFM reveals the distribution of charges and dipole‐carrying mechanochemical moieties, which directly correlates with changes in the work function.

The mechanochemical approach to forming charged species is shown in Figure 5. In short, mechanical strain and shearing forces increase in both sliding and tapping (contact‐separation) motions due to the mechanical and intermolecular forces between nano‐ and micro‐sized asperities on the metal surface and polymer chains at the interface. The bond rupture occurs under the condition where the potential energy of adhesion (adsorption) between polymer‐metal (*E_ads_*)^[^ ^74^ ^]^ and the potential energy of cohesion (*E_coh_*) between polymer bundles are equal in magnitude. *E_ads_* = *E*_*metal* − *polymer*_ −  *E_metal_* − *E_polymer_*. Here, *E_ads_* contributes to both physisorption and chemisorption in polymer‐metal surface interactions. It has been determined that the adsorption of PTFE oligomers on Cu surface is more favorable than the adsorption of PE oligomers, which indicates bond rupture, and triboelectric charging is more feasible for PTFE. Therefore, *E_ads_* is highly crucial for the metal‐polymer interfacial contact for sliding and separation electrification. *E_coh_* is the cohesive energy of a polymer,^[^ ^75^ ^]^ and it is equal to the sum of the three contributors. *E_coh_* = *E*_*coh* − *d*_ +  *E*_*coh* − *p*_ + *E*_*coh* − *H*_ here, d, p, and H represent dispersion, polar, and the H‐bonding components of the total cohesive energy, respectively. When the energy of adhesion between the polymer and metal, and the energy of cohesion, become equal in magnitude, stretching of polymer bundles across the counterfaces becomes feasible, and further extension of the polymer chain leads to bond rupture. If *E_ads_* it is not equal to *E_coh_*, pulling the polymer out of the bulk might occur, leaving the chain adhered to the metal surface, or leaving the chain on the polymer side without any bond rupture. Finally, bond scission occurs mechanochemically when the applied mechanical energy (*F*Δx) exceeds the bond's dissociation energy. Here, Δx is defined as the interatomic distance in the ruptured covalent bond. During the interfacial contact, we envision that the mechanical energy is transferred to the polymer chains. A clash of metal surface (or any other hard material) with nano‐ and micro‐sized asperities in a polymer causes bond scission in the polymer chains if the covalent bonds capture the mechanical compressing energy. During separation, the force captured by the covalent bonds stretched between the two counter surfaces leads to bond scission.

Based on the physicochemical requirements for the bond rupture (*E_ads_* vs *E_coh_*), and the force applied (*F*Δx) for bond rupture, the mechanism of the entire triboelectrification can be revealed by considering i) charge generation, ii) charge observation, iii) charge measurement, and iv) charge transfer processes separately. Therefore, it is necessary to divide the whole event into main parts. Each part has its characteristic multi‐processes and characteristic kinetics due to different reactions involved with varying energies of activation. For instance, the atmosphere affects the whole process differently since it exhibits different activation energies in different environmental conditions. The first primary process is the mechanochemical activation of covalent bonds either homolytically or heterolytically. The second process is the intra‐transformation of mechanochemical (+/−) charged polymeric fragments and radicalic end groups. The third related process involves external chemical reactions between, for example, H_2_O and O_2,_ and the mechanochemical generation of radicalic and (+/−) charged polymeric sites. The fourth and most crucial process is the electron transfer between metal and polymeric (+/−) charged fragments and radicalic sites, in forward or reverse direction, that takes place at the interface. These events are indicated in Figure 5, and labeled using the numbers from 1 to 4. The dynamic band energy model proposes mechanochemical bond rupture, the generation of polymeric fragments with (+/−) and radicalic sites, their possible intra‐transformation reactions, reactions of mechanoactive species with environmental reactants, and, finally, electron transfer reactions. Therefore, the mechanisms of charge generation and transfer are highly complex due to uncertainties in the identities and abundances of mechanochemical species, the rates of their transformation reactions, and their electronic interactions with the metal under experimental conditions.

Capturing mechanical energy by the covalent bonds in a polymer is a statistical event. The probability density of this event increases due to the stronger attractive forces during physical contact between the metal and the polymer. The mechanical energy that could have been concentrated in the covalent bonds excites them. Under the strain, the polymeric system undergoes a reactive strand extension, ultimately leading to bond rupture. When the applied mechanical energy (*F*Δx) that is absorbed by the covalent bonds exceeds the activation energy (*E_a_*) that is the energy required for bond rupture, then the bonds that are eventually "excited" start to break exponentially by the difference (*E_a_* − *F*Δx)/RT, either homolytically to give radicalic sites or heterolytically, yielding positively and negatively charged groups that bear localized dipoles. We believe that the covalent bonds of the polymer's structure determine the probability of bond rupture. For instance, polymeric fragments with (+/−) groups may not have been produced in PTFE, rather, radicalic sites could be formed and chemically transformed into e.g., peroxy radicals, hydroxy, and carbonyl groups in the presence of O_2_ and H_2_O. Nc,s is the number density of active sites contributing to charge accumulation at the polymer‐metal interface. The rupture of covalent bonds (*N*_*C*,*S*_) that are first mechanochemically activated and broken is a statistical event. When the local mechanical energy exceeds the intrinsic bond‐dissociation threshold, bonds break, generating active surface states that participate in the interfacial reactions and subsequently involved in the electron transfer to or from the metal.

(2) $$N_{C,S}=\int_{0}^{\infty }p\left(F\right)dFG\left(F\right)dF$$

where, *p*(*F*) is the probability density of ond rupture that captures the distribution of applied force *F*, with the strain. *G*(*F*) is the probability of the mechanochemical activation of the covalent bond under strain‐generated force. C and S refer to the contact and separation, respectively. *G*(*F*) depends on the force‐coupled rate constant *k*(*F*) of the bond rupture process and is related to mechanochemical activation as *k*(*F*) ≈  *e* ^−(Δ*Ea* − *F*Δ*x*)/*RT*^ at the given temperature.^[^ ^43^, ^78^, ^79^ ^]^ Here, bond rupture at the backbone of a polymer chain yields two polymeric fragments with primary active end groups.^[^ ^80^ ^]^ The equation highlights the fact that charge density is mainly determined by the activation energy of the bond rupture and the mechanical energy applied locally. If the difference between the activation energy and the rupturing force (*E_a_* − *F*Δx) and *p*(*F*) is high, then the triboelectric charge density or charge generation and the charge transfer by electrons will be the highduring contact‐separation and sliding motions. Enhancing the rate of bond rupture facilitates the electron transfer during the physical contact. In addition to the bond rupture that occurs between the atoms at the backbone chain, the second possible bond rupture can occur when an atom or side group is removed from the polymer backbone due to shearing forces. In this case, bond rupture may yield more stable secondary (2°) mechanochemically active sites on the polymer backbone. In that case, the reactions of the metal surface with an atom or a side group that is removed from the polymer backbone could be observed.^67^

Overall, the triboelectric charging process is initiated by mechanochemical activation of the covalent bonds (R─R), which have an activation energy of *E*_*a*, *mechano*_, generating mechanochemically active (R, R^+^, and R^−^) species on the polymer surface. Afterward, these reactive species react with environmental species such as O_2_ and H_2_O, potentially forming more stable radicalic products^[^ ^68^ ^]^ or transforming into charged or neutral products after structural rearrangement reactions. *E*_*a*, *external*_ represents the activation energy of all possible chemical reactions between external environmental reactants. Alternatively, these mechanochemically generated reactive intermediate species (R, R^+^, and R^−^) may react with each other via intra‐transformation reactions, yielding intermediates with various possible activation energies, represented by *E*_*a*, *intra*_ . Finally, mechanochemical species (R, R^+^, and R^−^) may form stable donor‐acceptor (D^+^A^−^) complexes or more stable anionic and cationic radicalic species bearing dipoles, as observed by KPFM. These species may be involved in the electron transfer mechanism.

The last primary process involves an electron‐transfer reaction that occurs in either the forward (metal to polymer) or reverse direction, or simultaneously in both directions, between the metal and mechanochemically generated surface states. This process has a characteristic activation energy, related to the work function of the metal, energies of the surface states, and the energy barrier of the polymer‐metal junction. The rate of electron transfer, therefore, depends on the energy match between the Fermi level of the metal and the polymer's mechanochemical surface states. These states are dynamically formed and decay through intra‐transformation reactions, external reactions, and their population density changes at each contact‐separation and sliding motion. Therefore, it is difficult to estimate their transformation rates under ambient conditions, since the activation energy of the electron‐transfer reaction in a dynamic, fast event involving such unstable species is unpredictable. $E_{a,e^{-}trans}$ represents the activation energies of electron transfer from metal to surface states of mechanochemical species (R, R^+^, and R^−^), or vice versa at the metal‐polymer interface. The transfer of charge by the electron transfer mechanism would be possible if the activation energy $E_{a,e^{-}\;trans}\;$ is comparable or smaller than either *E*_*a*, *intra*_ or *E*_*a*, *external*_. Otherwise, there would be no triboelectric charge transfer. The activation energies of electron transfer, $E_{a,e^{-}trans}$, depend on the electronic properties of the metal and interfacial species. The energy difference between the Fermi level of the metal and the energies of mechanochemically generated surface states determines the direction of the electron flow. For instance, charge transfer occurs from the polymer to the metal, and the polymer will be net positively charged if the Fermi level lies below the energy level of the singly occupied molecular orbital of the radical (SOMO), or the occupied energy levels of the surface states that correspond to the anionic/ radical anionic species. Likewise, charge transfer occurs from the metal to the polymer, and the polymer will be net negatively charged if the Fermi level lies above the energy level of the singly unoccupied molecular orbital of the radical (SUMO), or the unoccupied energy levels of the surface states that correspond to the cationic species and radical cationic ions. A recent theoretical study explored electron transfer mechanisms between metals and mechanochemically generated radical defect sites on PTFE, highlighting how the relative energies of donor‐ and acceptor‐like surface states determine both the direction and efficiency of triboelectric charge transfer.^[^ ^68^ ^]^

In light of these perspectives, we conclude that, although surface charge density and the charge transfer by electrons occur at the interface and they are closely related to each other, however, they are fundamentally different phenomena. The surface charge from charged species and dipoles generates a contact potential difference between these species and the metal probe, which is mapped using KPFM. The net surface charge density can be measured using a Faraday cup and a charge‐measuring device, such as an electrometer. Conversely, the transfer of charge by electrons is measured using an electrometer, and these electrons flow from a TENG device to be used as a power supply. To enhance the surface charge density, one needs to increase the rate of the bond‐breaking process by decreasing *E*_*a*, *mechano*_. Second, *E*_*a*, *external*_ should be kept high to decrease the rate of formation of more stable, e.g., oxygenated and neutral products. However, humidity is a critical parameter in electrification since H_2_O is known to stabilize the surface charges. The formation of either positively or negatively charged water clusters stabilizes the charged groups (R^+^ and R^−^) and increases the surface charge, and *E*_*a*, *external*_ should be low in that case. The intra‐transformation reaction rate of mechanochemically generated species (R, R^+^, and R^−^) should be low (high *E*_*a*, *intra*_) to allow the surface charges to persist longer. Lastly, the accumulation of charge on the surface is increased by maintaining a low electron transfer rate (high $E_{a,e^{-}trans}$). These effects suggest a reduced electronic interaction between the metal and the mechanoactive species, leading to charge buildup and higher surface charge density. On the other side of the coin, the triboelectric charge (electron) transfer between a metal and a polymer can be increased by controlling the rates of all the other pathways. This could be achieved by decreasing *E*_*a*, *mechano*_, increasing *E*_*a*, *intra*_, and increasing *E*_*a*, *external*_ to provide more charge flow and get more powerful generator devices. Enhancing triboelectric charge transfer by increasing electron flow rate can be achieved, e.g., through interfacial surface modification, as illustrated in many studies. On the other hand, the formation of positively and negatively charged water clusters (low *E*_*a*, *external*_), and stable anionic and cationic radical mechano‐ions (low *E*_*a*, *intra*_) enhances the inductive triboelectrical signal due to the electric field generated by these charged surface groups at the metal‐polymer interface. Since all possible processes are governed by their respective activation energies ($E_{a,e-\mathrm{mech}\mathrm{ano}},E_{a,e-\mathrm{trans}},E_{a,\mathrm{exte}\mathrm{rnal}},E_{a,\mathrm{intra}}$) and involve chemical reactions, they can be described by temperature dependent rates, modeled by Arrhenius‐type expressions. These rates include the homolytic bond rupture rate: $k_{\mathrm{homo},s,m}\left(T\right)$ ionic pathway rate: $k_{\mathrm{ionic},s,m}\left(T\right)$ secondary reaction rate: $k_{\mathrm{react},s,m}\left(T\right)$ ionization/polaron formation rate: $k_{\mathrm{ioni}\mathrm{ze},s,m}\left(T\right)$, and recombination rate: $k_{\mathrm{recmb},s,m}\left(T\right)$. The model equation describing the charge density from a mechanochemical perspective is provided in the Supporting Information.

Other semicrystalline polymers, including poly(ethylene) (PE), poly(vinylidene fluoride) (PVDF), and amorphous poly(vinyl chloride) (PVC), were employed to investigate the effect of stretching on their contact‐separation triboelectric charging behavior (Figure S11, Supporting Information). Strain decreased the contact tribocharging of PE. FTIR spectra indicate that some structural changes occur in the signals at 718 and 730 cm^−1,^ which are assigned to the ─CH_2_ rocking mode and reflect conformational and rotational motions of the polymer backbone (see the inset in Figure S11d, Supporting Information). Since PVDF has a high Young's modulus, the low strain did not change the mechanical properties of PVDF. However, we observed an increase in triboelectric charging of PVDF upon stretching, as shown in Figure S11g,h (Supporting Information). Main spectral changes in the IR spectrum occurred in the β‐phase that absorbs at 600 cm^−1^, the α‐phase at 795 and 855 cm^−1^ (due to CH_2_ out of plane rocking, C─C in phase symmetric stretching upon mechanical treatment. PVC is an amorphous polymer; therefore, its crystallinity and related mechanical properties remain unchanged by strain, as shown in Table S3 (Supporting Information). Therefore, the strain did not affect the triboelectric charging of PVC. Moreover, FTIR spectra of PVC did not indicate any significant structural changes (Figure S11n, Supporting Information).

## 3 Conclusion

Contact separation and sliding motions create dynamic interactions at the polymer‐metal interface, generating mechano‐activated species with distinct surface states that act as electron traps (acceptor) or sources (donors). These states are inherently transient, continuously forming and decaying, which dynamically alters the interfacial effective polymer work function. Our unified model, which incorporates the dynamic energy band alignment of mechanochemical surface states, captures the full spectrum of interfacial pathways, enabling both transient and steady‐state triboelectric charge accumulation to be predicted, and thus provides a comprehensive framework for understanding why different polymers exhibit distinct charging behaviors.

In this study, we first showed that structural changes in the polymers alter their work functions, as demonstrated by KPFM and UPS. UPS measurements of the stretched and unstretched PP and PTFE polymers are consistent with the triboelectric charging trend. Mechanical effects, such as uniaxial strain‐induced elongation of semicrystalline polymers, result in the cooperative, simultaneous evolution of amorphous and crystalline components, as demonstrated by XRD and FTIR analysis. Additionally, plastic deformation on the polymer surface was observed using SEM and AFM. EDX measurements indicated that the transfer of material from the polymer to the metal surface is an inherent part of the charging mechanism and that physisorption or chemisorption can alter the electronic energies of the interacting material pairs. Triboelectric charging experiments using stretched polypropylene showed increased charging in both contact and sliding modes. We observed that, compared to unstrained PP, strain‐induced polypropylene transferred less material to the SS metal surface. SEM analyses showed that PTFE polymer debris was deposited on the metal surface. As a result, triboelectric charging decreased over time due to the physisorption of the polymer onto the SS metal revealed by XPS measurements (Figure S8, Supporting Information); the interface exhibited polymer‐polymer interactions at the reciprocal surfaces. XRD measurements revealed that stretching alters the crystallinity of polymers, significantly affecting their mechanical properties. Both physical changes, such as strain hardening, and chemical structural changes, are considered the primary parameters that affect triboelectric charging. We changed the strain rate to reveal the effect of crystallinity on triboelectric charge generation. Under low strain rate polymer chains have more time to relax and reorient. Therefore, strain‐induced crystallization was promoted and triboelectric charge generation decreased for semi‐crystalline PTFE at low rates, Figure S6 (Supporting Information). Additionally, a rigid crystal structure causes less dipole reorientation and less polarization under low strain rate. At high rates, on the other hand, the mobility of the chains are high, and higher triboelectric charging because the amorphous regions provide more active and accessible sites for charge transfer. Due to the flexibility of chains, mechanochemical activation is promoted in amorphous regions. Contact‐separation and sliding mode of triboelectric charging of PTFE that are mechanically stretched at different ratios are shown in Figure S7 (Supporting Information). Starting from the first contact, electrostatically neutral stretched and unstretched PTFE samples show a gradual increase in contact‐separation triboelectric signals. Compared to the unstretched samples, the stretched ones are less tribocharged due to the loss of amorphous structures that generate more charge‐trapping mechanoactive sites and states.Mechanical work couples directly into the interfacial chemical bonds when a polymer surface slides or separates from a metal. Normal and shear forces distort and elongate these bonds, elevating their potential energy. Once the mechanical work per bond exceeds the intrinsic dissociation threshold, discrete bond scission events occur. Each rupture generates paired electronic defects (donor‐like and acceptor‐like states) whose asymmetry under metal‐polymer band alignment permits electron transfer while bond scission generates mobile radical fragments that further mediate charge movement depending on the relative work functions. In contact‐separation, normal‐force‐driven rupture produces localized charge patches that remain trapped in polymer surface states. The resulting surface charge density reflects the competition between: (i) mechanically driven bond breakage, (ii) energy‐aligned electron transfer, and (iii) relaxation pathways such as recombination or charge migration. This unified mechanism connects tribo‐mechanical work and interfacial electronic structure, explaining why materials with similar mechanical properties can exhibit markedly different triboelectric outputs.

Complete control over triboelectric charging can thus be achieved by tuning the polymer's physical, physicochemical, and electronic properties. Since triboelectric charge generation involves multiple mechanoactivated processes, each with its own activation energy, accurately predicting surface charge density requires accounting for the full spectrum of interfacial mechanochemical pathways and their dynamic surface states. Notably, both transient (time dependent) and steady‐state surface charge accumulation can be explained using the same model, underscoring its comprehensive applicability to triboelectric phenomena. Our model quantitatively captures the mechanistic origins of triboelectric charging and predicts trends across different polymers, yet absolute differences in charge accumulation also depend on surface heterogeneity, microstructural features, and environmental conditions, which introduce deviations beyond the model's deterministic scope. Lastly, our model accounts for key factors affecting surface charge density, and additional physical and chemical parameters‐ such as surface roughness, temperature, humidity, mechanical contact (including contact pressure, sliding‐friction, contact duration etc.), molecular orientation, chemical functionalities, molecular weight, and characteristic time constant of charge accumulation or decay‐ influence charging. Although some of these effects are indirectly accounted for through rate constants, considering them in future studies alongside mechanochemical events could further refine the understanding of triboelectric surface charge phenomena. Moreover, the differential mobility of charge bearing segments mediates how mechanochemically generated states migrate, coalesce, or remain trapped ultimately shaping the persistence and spatial distribution of interfacial charges. This requires a deep understanding of the mechanochemical surface states that form at the interface during dynamic contact, including not only their formation but also their subsequent transformations, environmental reactions, stabilization processes, and mutual interactions.

## Conflict of Interest

The authors declare no conflict of interest.

## Author Contributions

S.D.E. and H.T.B. wrote the manuscript. All authors have approved the final version of the manuscript. S.D.E. and H.T.B. conceptualized and developed the methodology, visualized the original draft, and wrote and edited the manuscript. Z.Y., T.D.C. mainly worked in the experimental section to obtain spectroscopic data, prepare samples, and conduct electrical measurements and characterizations. The authors declare no competing financial interest.

## Supporting information



Supporting Information

Supplemental Movie 1

Supplemental Movie 2

Supplemental Movie 3

Supplemental Movie 4

Supplemental Movie 5

## Data Availability

The data that support the findings of this study are available in the supplementary material of this article.
