# Supplementary material for: The Dynamic Energy Band Model of Contact‐Separation and Sliding Mode Triboelectric Charging of Polymers at the Metal‐Polymer Interface
Source: Adv Sci (Weinh). 2026 Mar 12;13(21):e17094. doi: 10.1002/advs.202517094 (PMC13073248; doi:10.1002/advs.202517094)
Supplement: Supplementary file 1 — Supporting Information [file ADVS-13-e17094-s002.docx]

**Supporting Information**

**The dynamic energy band model of contact-separation and sliding mode triboelectric charging of polymers at the metal-polymer interface**

Author Addresses

Sunay Dilara Ekim¹, Zelal Yavuz², Tuğba Demir Çalışkan^3^, Hande Güler^1^, H. Tarik Baytekin*^,^¹^,4^

¹ Department of Chemistry, Middle East Technical University, Ankara, 06800, Turkey
² UNAM – National Nanotechnology Research Center, Bilkent University, Ankara, 06800, Turkey
³ Chemical Engineering Department, Ankara University, Ankara, 06800, Turkey

^4^ Polymer Science and Technology Program, Middle East Technical University, Ankara, 06800, Turkey

*Corresponding author: Email: tarikbay@metu.edu.tr

**The PDF file includes:**

Model equation and parameters

Materials and Methods

Figs. S1 to S7

Table 1 and Table 2

Movies M1 to M5

References

**Materials and Methods**

“Commercially available poly(tetrafluoroethylene) (PTFE, DuPont Teflon), characterized by a melting temperature of ~330 °C, and poly(propylene) (PP), with a melting temperature of ~162 °C, were used in this study.” For the triboelectric charging experiments, 5 cm x 5 cm polymer films were attached to the grippers of the mechanical stretching device and stretched from 4.0 cm to 28.0 cm for the PP film and from 5.0 cm to 8.5 cm for the PTFE film before reaching the breaking point. The rate was 1 mm/s during the extension of PP, PTFE, and all other polymer films. SS metal beads were purchased from Ozrulman Company. SEM images were obtained using QUANTA 400F Field Emission SEM. ATR FTIR spectra were obtained using a Bruker Alpha-II FTIR spectrometer. XRD measurements were done using Rigaku Ultima-IV X-Ray diffractometer. Hardness measurements were done using CSM instruments mechanical tester. Carl Zeiss POM was used to observe alterations in PP's voids and crystalline domains during mechanical treatments. AFM height and KPFM surface charge mapping were done using Nanosurf FlexAFM system.

Tapping experimental setup

A homemade tapping device (Figure S7a and S7b, and Movie S4) was used to monitor the contact-separation triboelectric charging of polymers. The thickness of the PP film was 50 μm before the stretching and ~5.0 μm after the stretching. The thickness of the PTFE film was 60 μm before the stretching and ~50 μm after the stretching. A mechanical stretching device stretched 5cm x 5cm size unstretched PP and PTFE polymer films. The polymer sample was placed on an 18 mm-diameter disc-shaped aluminum stub. The polymer sample on the Al stub was tapped using a 5-mm-diameter disc-shaped copper stub at a 5 Hz frequency (Movie S4). Both metal stubs were polished using a SiC grinding paper (#1200), rinsed with deionized water and ethanol. The PP, PTFE, and other polymer thick film samples were placed onto the Al metal stub using a ring-shaped HDPE holder that keeps the polymer film flat and smooth on the metal stub surface, providing direct contact between the polymer film and the electrode surface. (Figure S7a) The use of adhesive carbon tape was avoided due to the differences in viscoelasticity between stretched and unstretched polymer films, as adhesive tape can cause varying amounts of impact forces during tapping. The open circuit electrical potential (in volts, V_OC_) is recorded by an oscilloscope (OWON XDS 3104E) during the contact and separation of the polymer film and copper surfaces. Oscilloscope probes with 100 megohm input impedance were connected directly to the copper and aluminum metal stub electrodes using a small brass piece. Standard deviations were calculated from at least five independent measurements. Relative humidity was measured as 23–45% during the measurements at room temperature.

Sliding experimental setup:

A homemade sliding contact device (Figure S1c and S1d, and Movie S5) is used to monitor the sliding triboelectric charging of polymers. Electric potential (V_OC_) was collected and recorded by an oscilloscope, where a 3.5 mm diameter stainless steel (SS) bead was connected to the probe using a small brass bead holder. The normal load applied to the bead is measured as 0.40 N using a pre-calibrated force-sensitive resistance. Unstretched and stretched polymer samples were placed on a flat metal stainless steel surface. Polymer samples were firmly attached to the flat sliding stage from its edges using conductive copper tape. The metal bead was slid parallel to the stretching direction of the polymer sample. The metal bead and brass holder were isolated from the other parts of the device using a wood insulator. The reciprocating motion at 5.0 Hz frequency of the sliding stage is controlled by a stepper motor. The triboelectric signal is recorded by an oscilloscope (OWON XDS 3104E). High-input-impedance (100 megohms) probes are used in the measurements. Relative humidity was measured as 23–35% during the measurements at room temperature.

Surface potential mapping:

KPFM surface potential mapping is performed by measuring contact potential difference (CPD) between the tip and polymer (PP, PTFE, and PE) surfaces, which were obtained using Nanosurf FlexAfm. Mapping of CPD obtained at each 256 pixels along a line gives the 2D surface potentials of the whole area. Surface potential is obtained with a conductive cantilever (PPP-MFMR, force constant is ~2.8 N/m) cantilever) at 100 nm height from the surface using amplitude mode KPFM. Polymer samples were attached to 1-mm-thick metal discs using a round-shaped conductive carbon tape.

XPS and UPS measurements:

A Mg Kα (hν = 1253.6 eV) light source (PHI 5000 VersaProbe) for the XPS and a monochromatized He I (hν = 21.2 eV) light source were used for the UPS measurements, respectively. The work function was evaluated from the cutoff energy of the UPS spectrum. A sample bias of 7V was applied to the polymer samples.

**Unified time-dependent triboelectric charging model for polymers: homolytic, heterolytic, adhesion and environmental effects, and time-dependent activations:**

$$\sigma\left( t \right)=\frac{1}{A}\sum_{s\in S} \sum_{m\in M_{s}} n_{e}\left( s,m \right)\text{ }w_{s,m}\text{ }f_{s,m}^{\text{mode}}\text{ }f_{s,m}^{\text{ionic}}\text{ }f_{s,m}^{\text{homo}}\text{ }f_{s,m}^{\text{react}}\left( t \right)\text{ }f_{s,m}^{\text{ionize}}\left( t \right)\text{ }f_{s,m}^{\text{recomb}}\left( t \right)\text{ }$$

$$H(F_{\text{mode}}\Delta x-E_{\text{bond}})\text{ }H(\Delta_{\text{ad}}-\Delta_{\text{crit}})$$

Time-dependent activation:

$$f_{s,m}^{\mathrm{react}}(t)=1-exp[-(k_{homo,s,m}+k_{ionic,s,m}+k_{react,s,m}+k_{ionize,s,m})t]$$

This formulation captures the full microscopic, time-dependent dynamics of surface charge, including contributions from existing ionic states, newly generated ions, homolytic and mechanochemical processes, activation and recombination kinetics, and environmental/mechanical thresholds.

**Parameter Descriptions**

- $n_{e}(s,m)$: Number of electrons available in a state $s$and in energy level $m$.
- $\sigma\left( t \right)=\frac{1}{A}\sum_{s\in S} \sum_{m\in M_{s}} \ldots\sigma\left( t \right)=$ Surface charge density
- Surface-region index $s\in S$. Here, $s\in\mathcal{S}$: species set; $\mathcal{S}$ ={"neutral"," radical"," anion"," cation"}
- $S$ denotes the set of all surface regions. The summation $\sum_{s\in S}$accounts for contributions from each surface region $s$to the total surface charge $\sigma(t)$.
- Micro-unit index $m\in M_{s}$:
- $M_{s}$denotes the set of all micro-units m within the surface region $s$. It is the energy levels associated with each species, energy levels associated with each species (HOMO/LUMO-generated, SOMO/SUMO, and polaron levels)
- The summation $\sum_{m\in M_{s}}$ensures that contributions from each micro-unit $m$ in the region $s$ are included in $\sigma(t)$.
- $w_{s,m}$: Reflects density of available mechanochemical states, and represents the relative contribution of each electronic state (SOMO/SUMO, ionic, or HOMO-like levels) to the overall triboelectric charge density.
- $f_{s,m}(\mathrm{mode})$: Fraction participating under a mechanical deformation mode describing how normal, shear, or mixed mechanical loading populates interfacial electronic states.
- $f_{s,m}^{\mathrm{ionic}}$: Represents the contribution from pre-existing ionic states (polarons, anions, cations, etc.) at micro-unit $m$ in surface region $s$. ionic/homolytic fractions. It denotes the ionic pathway contribution (ion-mediated or DA-complex–mediated activation). Optional contribution from ionic species (polaron/anion/cation)–derived states due to heterolytic bond rupture.
- $f_{s,m}^{\mathrm{homo}}$: Fraction contribution from homolytic bond rupture (SOMO/SUMO-like states).
- $f_{s,m}^{\mathrm{react}}(t)$: Time-dependent activation o**f reactive species,** and
- $f_{s,m}^{\mathrm{react}}(t)$ =$(k_{\mathrm{homo}}+k_{\mathrm{ionic}}+k_{\mathrm{react}}+k_{\mathrm{ionize}})$.
- $f_{s,m}^{\mathrm{recomb}}(t)$: Time-dependent recombination fraction for the micro-unit $m$ in the surface region $s$.
- $f_{s,m}^{\text{recomb}}\left( t \right)=1-\exp\left( -k_{\text{recomb}}t \right)$
- $H(F_{\mathrm{mode}}\Delta x-E_{\mathrm{bond}})$: Heaviside controlling bond rupture; 1 if mechanical work exceeds bond energy, 0 otherwise. It gives the adhesion threshold.
- $\Delta_{\mathrm{ad}}$, $\Delta_{\mathrm{crit}}$: adhesion/stretching threshold condition
- $H(\Delta_{\mathrm{ad}}-\Delta_{\mathrm{crit}})$: Adhesion condition and threshold; 1 if the polymer chain is sufficiently stretched and the interface distance increased, 0 otherwise.
- $F_{\mathrm{mode}}$: Local mechanical force along the deformation mode responsible for bond rupture (N; $F\cdot\Delta x$in J).
- $A$: Effective contact area.
- $F_{\text{mode}}$ local mechanical force along the deformation mode responsible for bond rupture
- $F_{\text{mode}}\Delta x$: local mechanical work (units: N; $F\cdot\Delta x$gives energy in J).
- $E_{\text{bond}}$: bond dissociation energy.

| $\Delta x$ | Bond extension/stretch distance (m) |
| --- | --- |
| $\Delta_{ad}$ | Polymer–metal adhesion induced chain extension (m) |
| $\Delta_{crit}$ | Critical distance for bond rupture (m) |
| $E_{bond}$ | Bond dissociation energy (J) |
| $H(F_{\text{mode}}\Delta x-E_{\text{bond}})$ | Heaviside function controlling bond rupture: 1 if mechanical  work exceeds bond dissociation energy, 0 otherwise |
| $H(\Delta_{\text{ad}}-\Delta_{\text{crit}})$ | Adhesion condition: 1 if polymer chain is sufficiently stretched and interface distance increased, 0 otherwise. |

**Rate Constants** $\boldsymbol{k}_{\mathbf{...}\boldsymbol{,s,m}}\boldsymbol{(T)}$**(s⁻¹)**

Rate Constants $k_{...,s,m}(T)$ All in s^-1^, with Arrhenius-like temperature dependence and optional multiplicative factors for mechanical work, relative humidity, or electric-field–induced barrier lowering.

$k_{\text{homo},s,m}(T)$ homolytic bond-rupture rate
Rate at which mechanochemical homolytic bond scission generates radical (SOMO/SUMO-like) surface states. Typically modeled with an Arrhenius term modulated by a mechanical-work function $g_{\text{mech}}(F_{\text{mode}}\Delta x)$.

$k_{\text{ionic},s,m}(T)$ ionic pathway rate
Creates the initial charged species. Mechanochemical activation rate of ion-mediated pathways (ion transfer, donor–acceptor complex–assisted ET, humidity-stabilized ions). Can be expressed as an Arrhenius term multiplied by a humidity-dependent factor $h(\text{RH})$.

$k_{\text{react},s,m}(T)$ secondary reaction rate
Rate describing chemically driven secondary surface reactions (oxidation, stabilization, formation or loss of surface states) initiated by mechanochemical activation. Depends on $T$ $\text{RH}$, and the ambient composition.

$k_{\text{ionize},s,m}(T)$ ionization/polaron formation rate
Rate for the formation of polymeric anions, cations, or polarons following electron transfer to or from the mechanoradicals.

$k_{\text{recomb},s,m}(T)$ recombination rate
Represents charge neutralization or charge recombination pathways, and it may be included when the fast decay of activated species (such as the charge decay of the PE and PDMS) is experimentally observed. birden fazla mekanizma varsa toplam olarak:

$$k_{\mathrm{rec},s,m}=k_{\mathrm{recomb},s,m}+k_{\mathrm{backreact},s,m}+k_{\mathrm{neutralize},s,m}$$

$$f_{s,m}^{\mathrm{react}}(t)=1-\exp[-(k_{\mathrm{prod}}-k_{\mathrm{recomb}})t]$$

- $k_{\mathrm{homo},s,m}(T)$: Homolytic bond rupture, generating radical states.
- $k_{\mathrm{ionic},s,m}(T)$: Ionic pathway; formation/activation of charged localized states.
- $k_{\mathrm{react},s,m}(T)$: Secondary chemical reactions (oxidation, side reactions, stabilization).
- $k_{\mathrm{reco}\mathrm{mb},s,m}(T)$: Recombination rate constant.
- $k_{\mathrm{ionize},s,m}(T)$: Ionization generates: polaron, anion, and cation.
- $k_{\mathrm{eff},s,m}(T)=k_{\mathrm{prod},s,m}+k_{\mathrm{rec},s,m}$ , and the time constant that describes how fast a given surface site–mode pair $(s,m)$approaches its steady charge state, $\tau_{s,m}=\frac{1}{k_{\mathrm{eff},s,m}}$

Small $k_{\mathrm{prod}}+k_{\mathrm{rec}}$ → large $\tau$→ long-lived, quasi-static surface charge

**Large** $k_{\mathrm{prod}}$ and **large** $k_{\mathrm{rec}}$ → small $\tau$→ rapidly evolving, dynamic surface charge

**Notes:**

**Note-1**: Ionic species already present in the material can give rise to localized energy states, appearing as additional energy levels. These states may be optionally included in the summation of total charge density contributions to the system.

**Note-2**: While the proposed model robustly captures both transient and steady-state triboelectric charge accumulation, several practical factors may influence its predictive accuracy. Surface morphology and real contact area can deviate from idealized assumptions, affecting local bond rupture and electron transfer. Environmental conditions, such as relative humidity and temperature, as well as Coulombic ‘‘back‘‘-repulsion at high charge densities, may further modulate surface charge. Additionally, the heterogeneous nature of polymers, the dynamic orientation of donor–acceptor complexes, and long-term wear or oxidation of surfaces could introduce some deviations from the model predictions.

**Note-3**: Although ionic (polaron/anion/cation) species have not been directly confirmed here, they may introduce localized frontier-like states that could participate in interfacial electron transfer; we therefore consider their potential role in the model as an extension. We explicitly include ionic species–derived electronic levels (polaron/anion/cation states) in the summation over interfacial states, since ionization shifts and localizes frontier orbitals and these charge-bearing species can directly participate in electron transfer to/from the metal.

**Note-4**: Each rate constant $k_{...,s,m}(T)$follows an Arrhenius-like dependence on temperature and may include additional multiplicative terms to account for mechanical work, relative humidity, or electric-field–induced barrier lowering. All the rate constants have s^-1^ units.

**Note-5**. The participation of each interfacial electronic state in electron transfer depends on its energy relative to the metal’s Fermi level. Only states aligned or sufficiently close to the Fermi level can effectively contribute to triboelectric charging. This can be incorporated via an *alignment factor* $f_{s,m}^{\mathrm{align}}$, e.g., a Heaviside function $H(E_{s,m}-E_{F})$ or a smooth sigmoid $1/(1+\exp[(E_{s,m}-E_{F})/k_{B}T])$, which selectively weights the contribution of accessible states in the summation over interfacial levels.

**Note-6**. Adsorption-Induced Dipole Layer and Charge Decay Mechanism: Repeated contact–separation and sliding cycles lead to a gradual decrease in the triboelectric output of PP and PTFE. This decay originates from the progressive formation of an adsorption-induced interfacial dipole layer on the stainless-steel surface. Mechanochemically detached polymer fragments transfer onto the metal and subsequently physisorb or chemisorb, forming a thin overlayer. XPS measurements (Figure SI8) confirm this material transfer through the appearance of PTFE- and derived chemical signatures on the SS surface. The adsorbed fragments generate a Helmholtz-type interfacial dipole, which modifies the local vacuum level and shifts the effective metal–polymer energy alignment. The Helmholtz equation describes the corresponding potential shift:

$\Delta V={\mu_{\text{ad}}/\varepsilon}_{0}A$

$$\sigma=\varepsilon_{\text{o}}\Delta V/d$$

$$\sigma=\mu_{\text{ad}}/Ad$$

where,
$\mu_{\text{ad}}$= net dipole moment of the adsorbed layer,
$A$= interfacial area,

$\sigma$ = surface charge density (C/m²),

$d$= thickness of the dipole layer (m),
$\varepsilon_{0}$= vacuum permittivity.


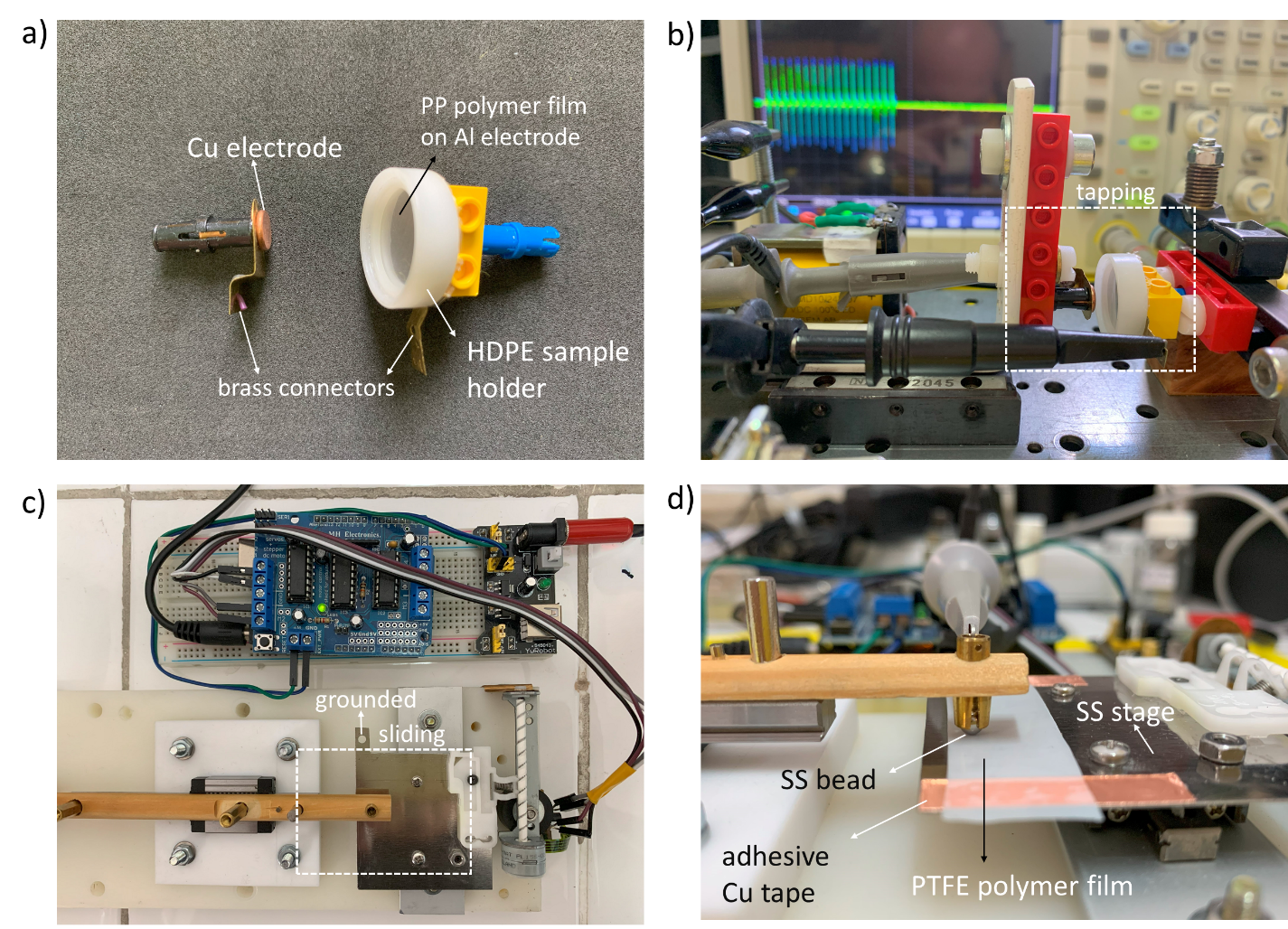


**Figure S1.** Photographs showing: a) Copper electrode, PP polymer on Al stub electrode and the sample holder, b) tapping device, c) set-up of sliding triboelectrification, d) PTFE film sample placed on stainless steel sample stage, shown in (c).

**
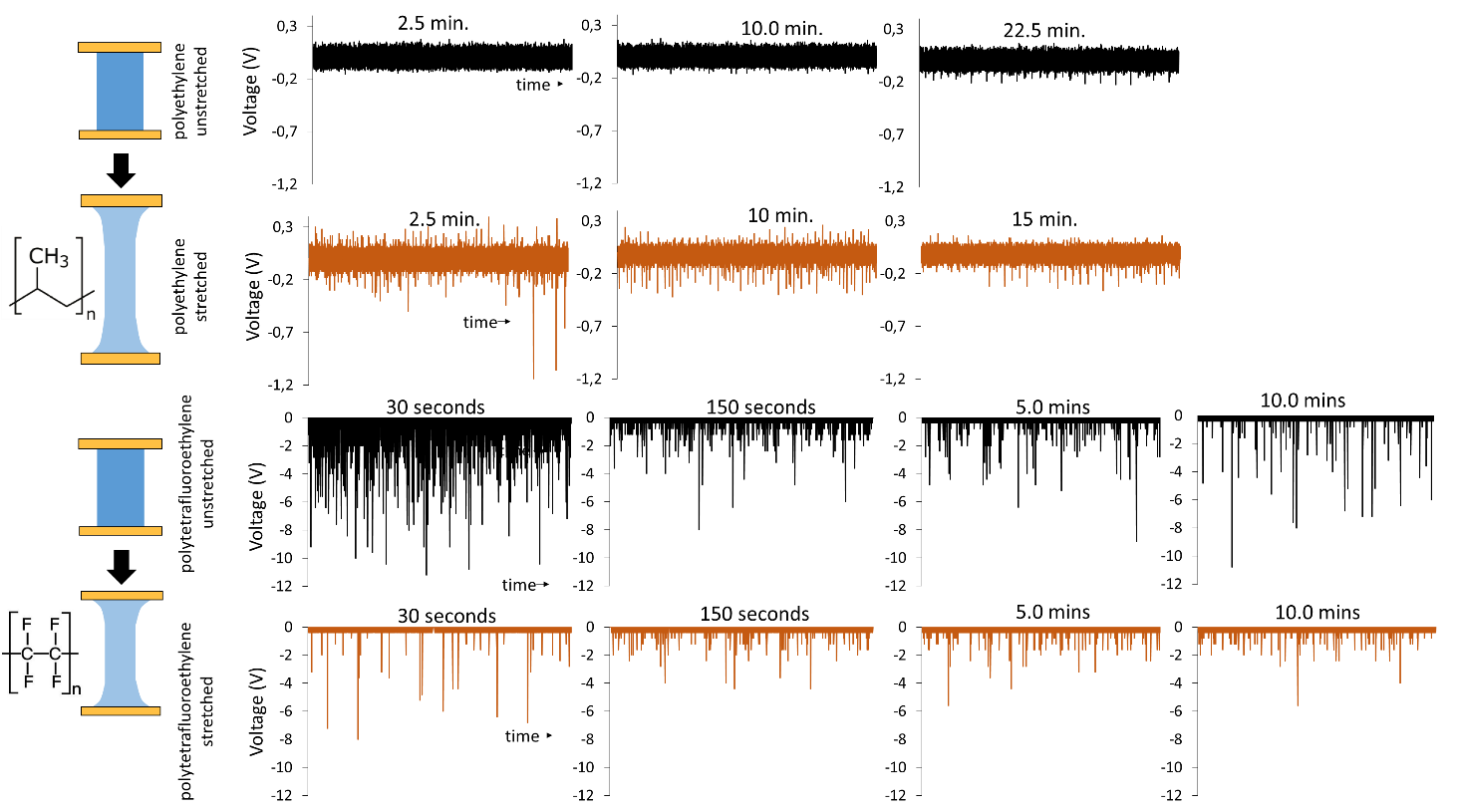
**

**Figure S2.** Sliding mode triboelectrification signals collected from the metal bead connected to the probe, and the bead is contacted and slid on the polymer PP and PTFE films. The total time of sliding are indicated on top of each triboelectric voltage vs. time plots. Sliding triboelectrification of j) unstretched PP film, k) unstretched PP film, and l) unstretched PTFE film, m) unstretched PTFE film). RH=30%, T= 25^o^C. A heterogeneous charge distribution was observed in the friction of PP with a metal. ^1^

**
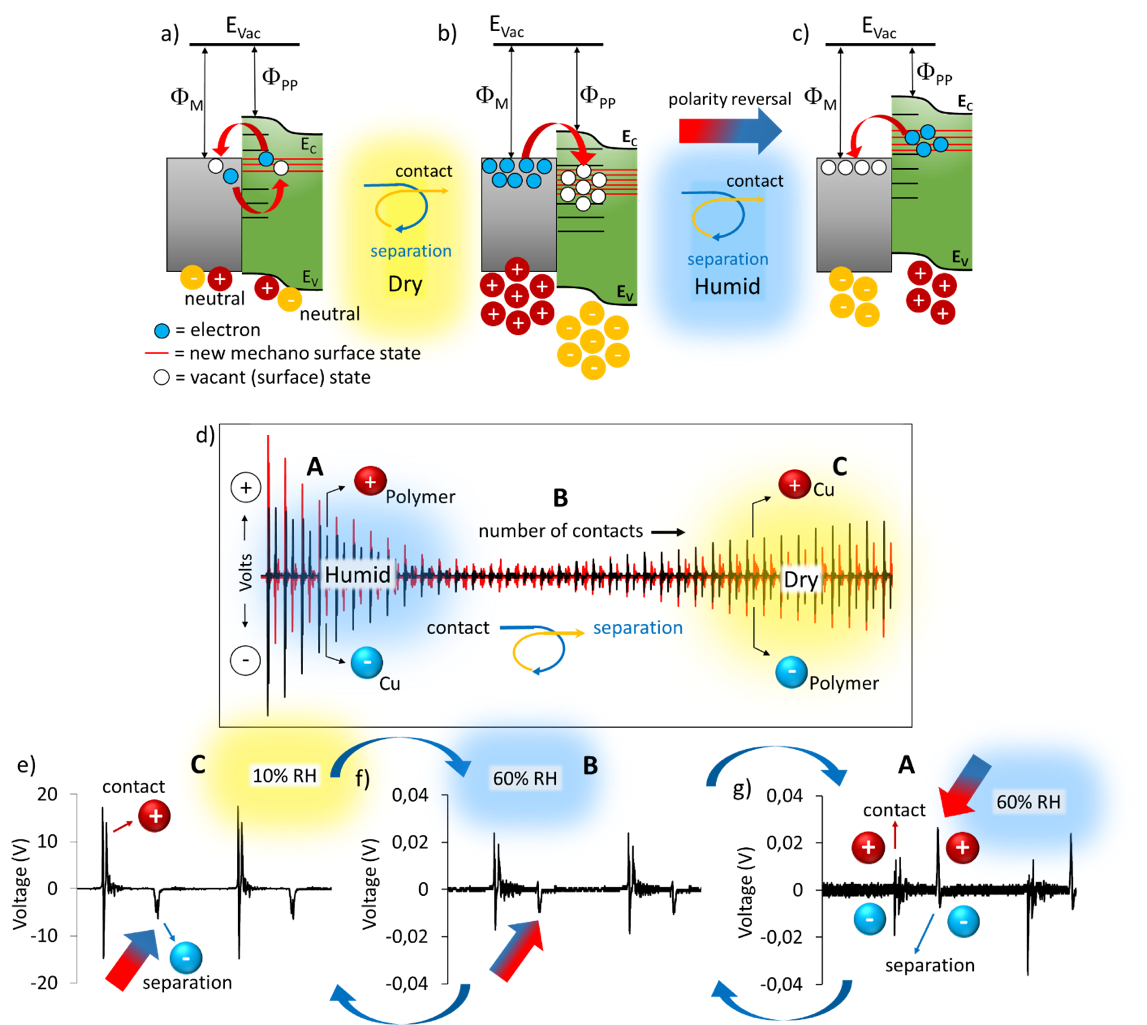
**

**Figure S3.** Bipolar charging at the contact and the polarity reversal observed in separation electrification for PP film. a) and b) the increasing number of mechanoactivated surface states during contact-separation cycles under dry atmosphere conditions. b) during the contact under low humidity the charge transfer occurs from metal to polymer, and the polymer is charged negatively under a dry atmosphere (RH≤10%) after separation. c) during the contact under a high humidity (RH≥50%), where the polymer surface is deposited with water molecules, charge transfer is reversed after the separation. d) contact-separation triboelectric signals that show a positively charged polymer under high humidity, RH≥50% gets negatively charged under dry atmosphere conditions (RH≤10%). e) contact-separation electrification cycles (two cycles were shown) show polarity reversal from (-) to (+) of the separation signal (shown in (A)) when the humidity is changed instantly from RH=10% to 50 % by purging humid air onto the polymer during tapping. Charge density (σ) and RH relationship can be estimated using the equation:


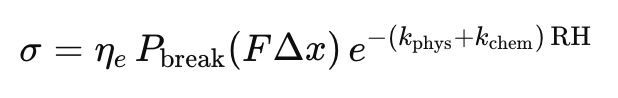


Here, $\eta_{e}$ is the effective number of electrons that actually escape after a bond rupture, $P_{\mathrm{break}}(F\Delta x)$ is the bond-breaking probability that shows the probability that the applied mechanical work (normal or shear) exceeds the bond dissociation energy. $F\Delta x$is the local mechanical work portion of applied mechanical energy delivered directly to nanoscale interfacial bonds. RH is the relative humidity; $k$is a material-specific sensitivity coefficient describing the exponential decay of charge density with increasing RH. Hydrophobic materials (e.g., PTFE) have small $k$, while hydrophilic or hydrogen-bonding polymers have larger $k$. The “0–1” range represents the fractional surface moisture, where 0 corresponds to a completely dry surface and 1 corresponds to a surface fully covered by water. This fraction scales the effect of physical ($k_{\text{phys}}$) and chemical ($k_{\text{chem}}$) screening, indicating how much the surface water and ionic or radical interactions contribute to the reduction of accumulated charge.; higher RH increases both chemical and physical screening. $k_{\mathrm{phys}}$ is the physical screening coefficient that represents charge reduction due to water layers and ionic films that screen electrons and ions. $k_{\mathrm{chem}}$ is the chemical quenching coefficient that represents the effect of radicals or reactive surface groups interacting with water (radical quenching, proton transfer, generation of radicalic (ionic) species, etc.).


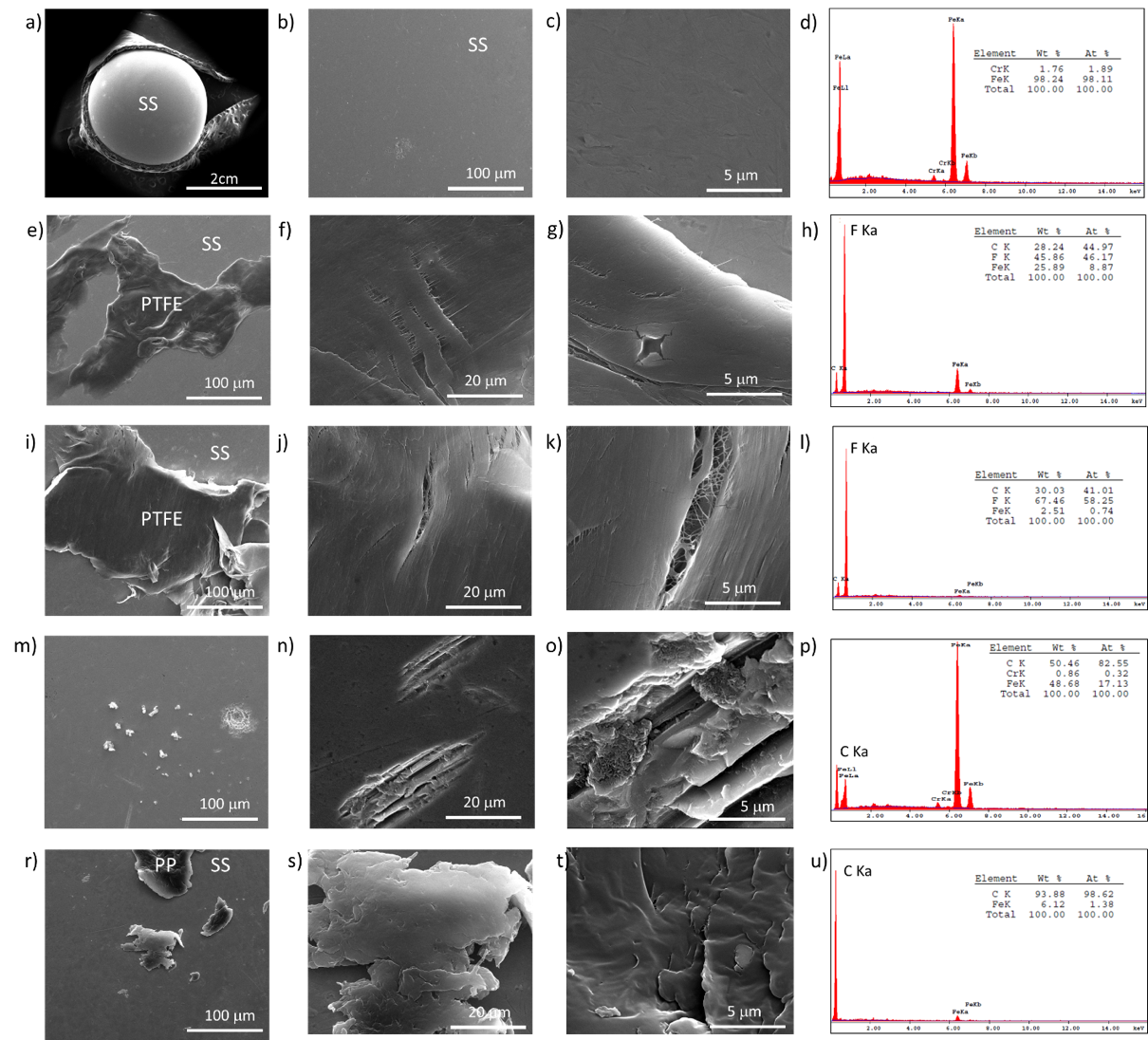


**Figure S4.** SEM images of SS metal beads that indicate the transfer of polymer to the rubbed surface of the SS bead. a) to c) SEM images of clean and unrubbed SS bead surface, and d) EDAX elemental analysis of the bead surface before sliding. Elemental compositions are given in the table as insets in EDAX spectra. e) to g) SEM images of rubbed SS bead to **unstretched** **PTFE** after sliding, and h) EDAX elemental analysis of the bead surface after sliding. i) to k) SEM images of rubbed SS bead to **stretched** **PTFE** after sliding, and l) EDAX elemental analysis of the bead surface after sliding. m) to o) SEM images of rubbed SS bead to **unstretched** **PP** after sliding, and p) EDAX elemental analysis of the bead surface after sliding. r) to t) SEM images of rubbed SS bead to **stretched** **PP** after sliding, and u) EDAX elemental analysis of the bead surface after sliding.


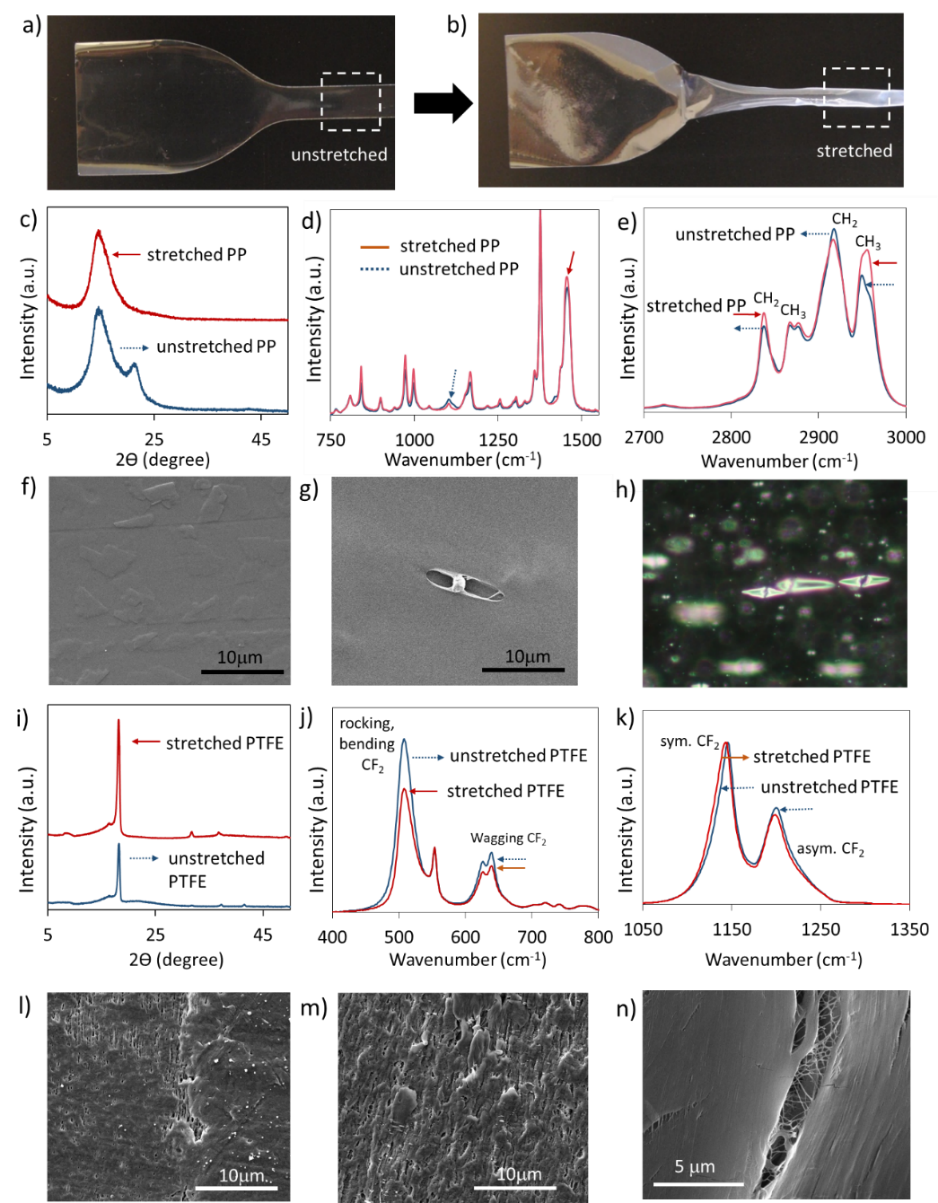


**Figure S5.** Optical photographs, XRD diffractograms, SEM images, and FTIR spectra showing structural and morphological changes upon uniaxial stretching of PP^35^ and PTFE thick films. Photographic images of unstretched (a) and stretched (b) PP films show a clouding effect upon stretching. XRD diffractogram indicates the physical structural changes in PP film (c) and in PTFE^36^ (i) due to the stretching. A decrease in crystallinity is accompanied by the disappearance of the signal at 21.6 degrees in the diffractogram of the mesomorphic form of the PP film (Figure 5c). ATR-IR analysis reveals the changes in the chemical structure of the PP film upon stretching (d and e) and for PTFE (j and k)^2,3^. Spectra are normalized at 1375 cm^-1,^ and the peak is assigned to the symmetric bending vibration mode of the CH_3_ group for PP. SEM images showing stretch-induced surface morphological changes in PP film (f and g), and in PTFE (l and m). Polarized optical microscopy image of the stretched PP film indicates the formation of voids that cause a cloudiness effect upon stretching (h). n) SEM image of PTFE film transferred to the contact surface of SS bead after sliding the bead on stretched PTFE, see Figure S4. ATR-IR spectra of the strain-induced changes in the chemical structure in PTFE films. The ATR-FTIR spectrum of PTFE shows two strong peaks at 1151 cm^-1^ and 1210 cm^-1^ which could be attributed to the symmetric and asymmetric stretching vibration of the molecular chains of CF_2_.^2^ The rolling vibrations γω (CF_2_) appear at 639 cm^–1^, 555 cm^–1,^ and 516 cm^–1^ is due to deformation of -CF_2_ groups.^2,3^

^
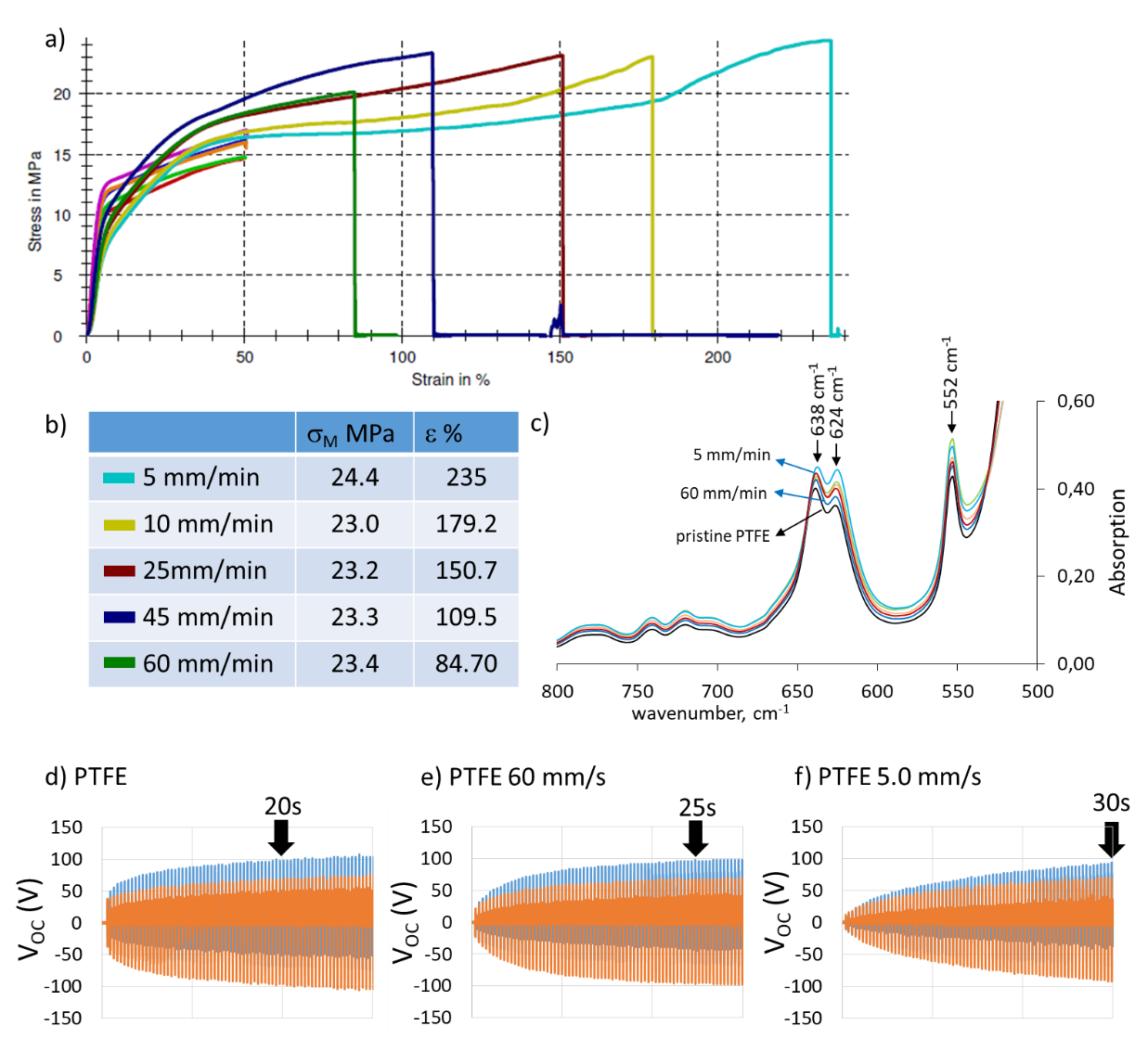
^

**Figure S6.** a) Mechanical testing of PTFE samples stretched at different rates. b) modulus and percent extension of the samples, PTFE polymer films were stretched at different elongation rates (given in (b)) until the rupture point. c) FTIR spectra of the PTFE^3^ samples show an increase in absorption peaks at 624 cm^-1^, 638 cm^-1^, and 552 cm^-1^. In particular, the band at 625 cm^−1^ was supposed to be correlated with the“defect” occurring in the polymer chain. ^4c^ and the effect of stretching rate is more visible when the signal at 638 cm^−1^ is normalized (not given). d) Contact-separation triboelectric charging of PTFE film attached to an Al metal electrode using a conductive carbon tape. Samples were rinsed and neutralized using ethanol before charging. RH: 35%, T= 25^o^C.

**
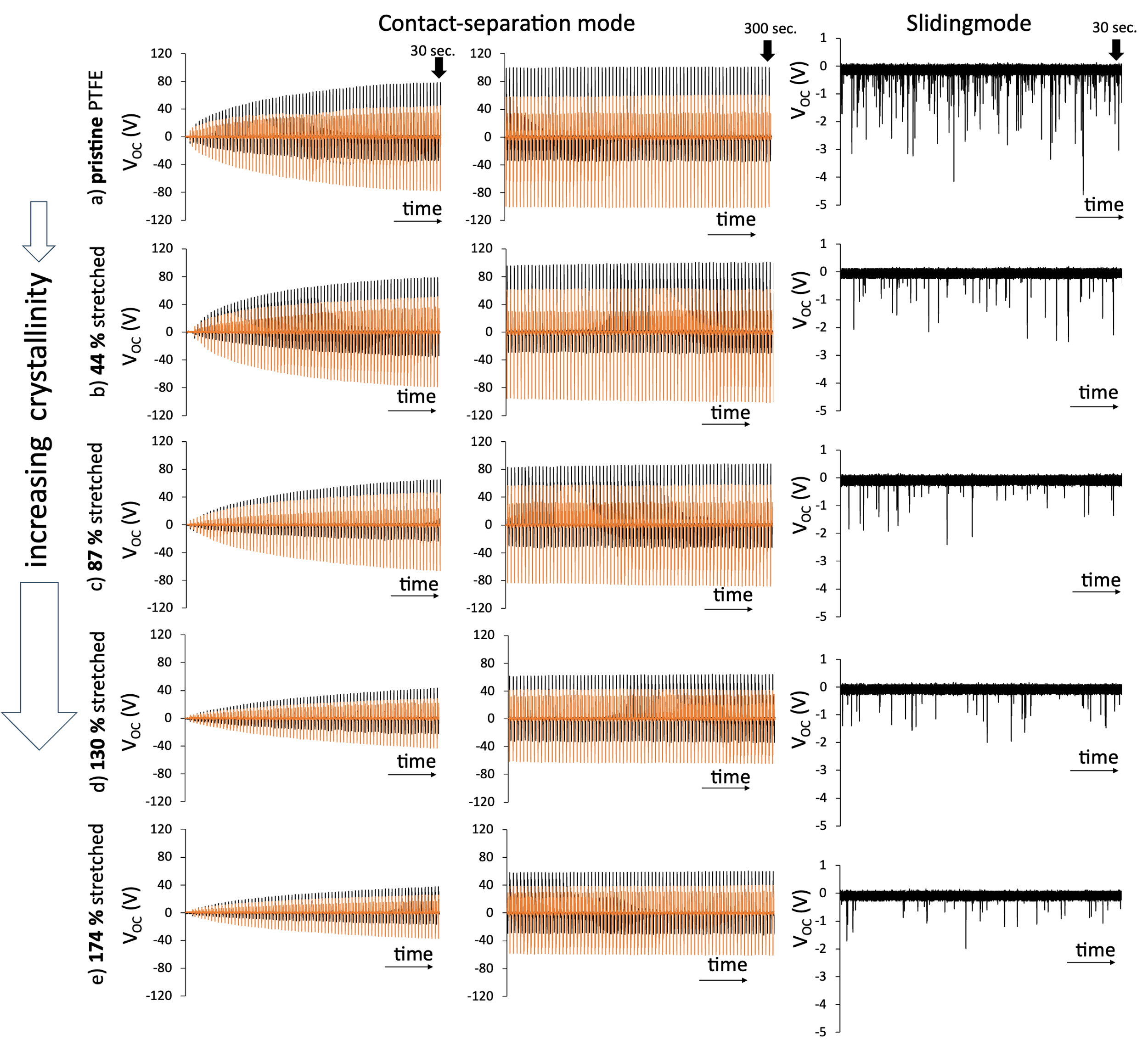
**

**Figure S7.** From left to right: Contact-separation mode of triboelectric charging of PTFE that is mechanically stretched at different ratios. Triboelectric charging was obtained in contact-separation mode using a tapping device and an oscilloscope system (left). Triboelectric signals after 5 minutes (middle). Sliding mode of triboelectric signals obtained from the sliding device (right). Triboelectric signals decrease for the PTFE samples that are subjected to 174% elongation. Starting from the first contact, electrostatically neutral samples show a gradual increase in contact-separation triboelectric signals. The rise in signals is attributed to the increase in the density of mechanochemical surface states after each contact-separation cycle (see the main text). PTFE film ruptured after 215% elongation. RH: 42%, T= 25^o^C. XRD diffractograms indicate the increase in crystallinity.

**
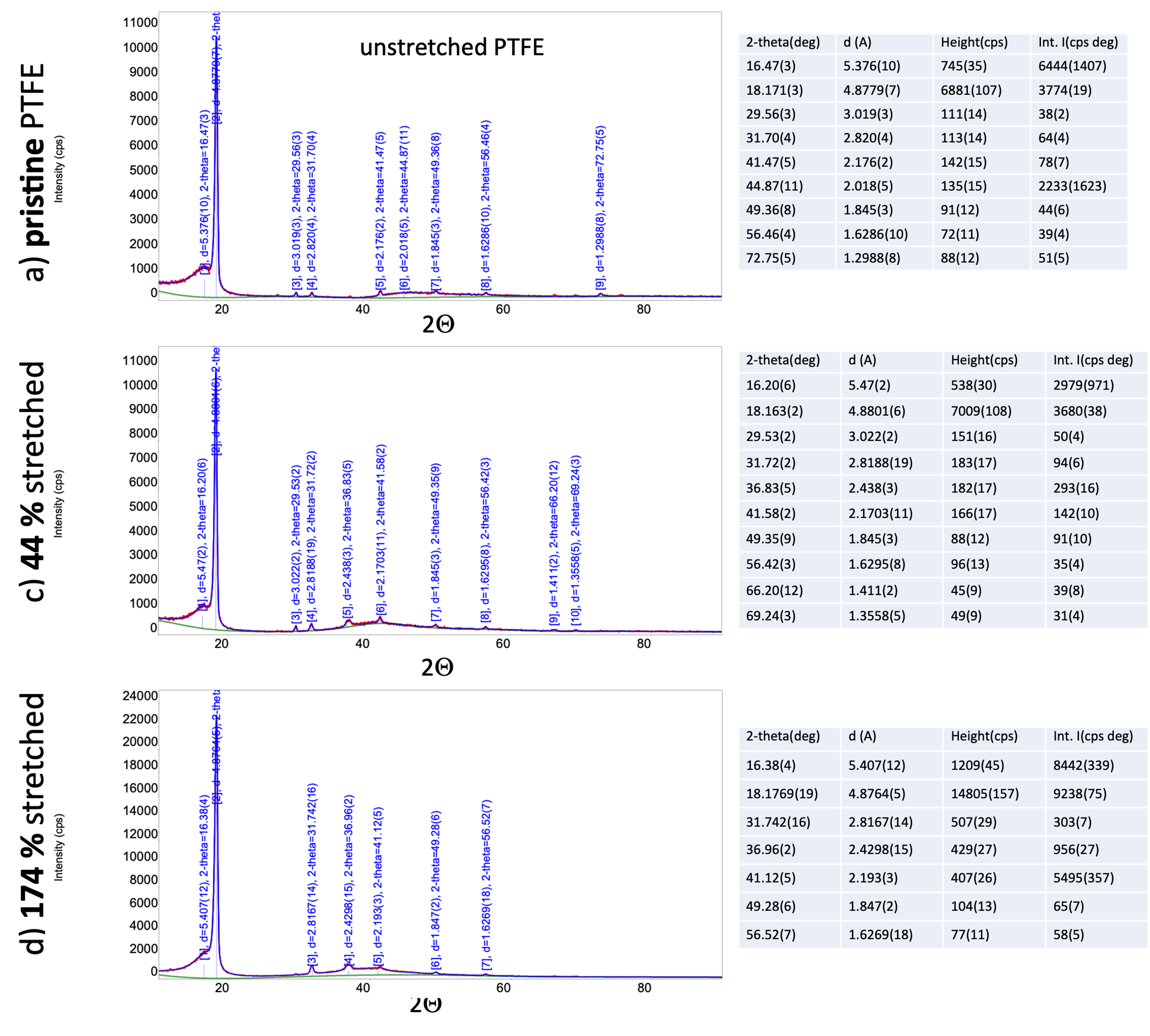
**

**Figure S7. Continued…** XRD diffractograms of the PTFE films that were stretched at different ratios (44% and 174%) indicate the increase in crystallinity upon stretching.

**
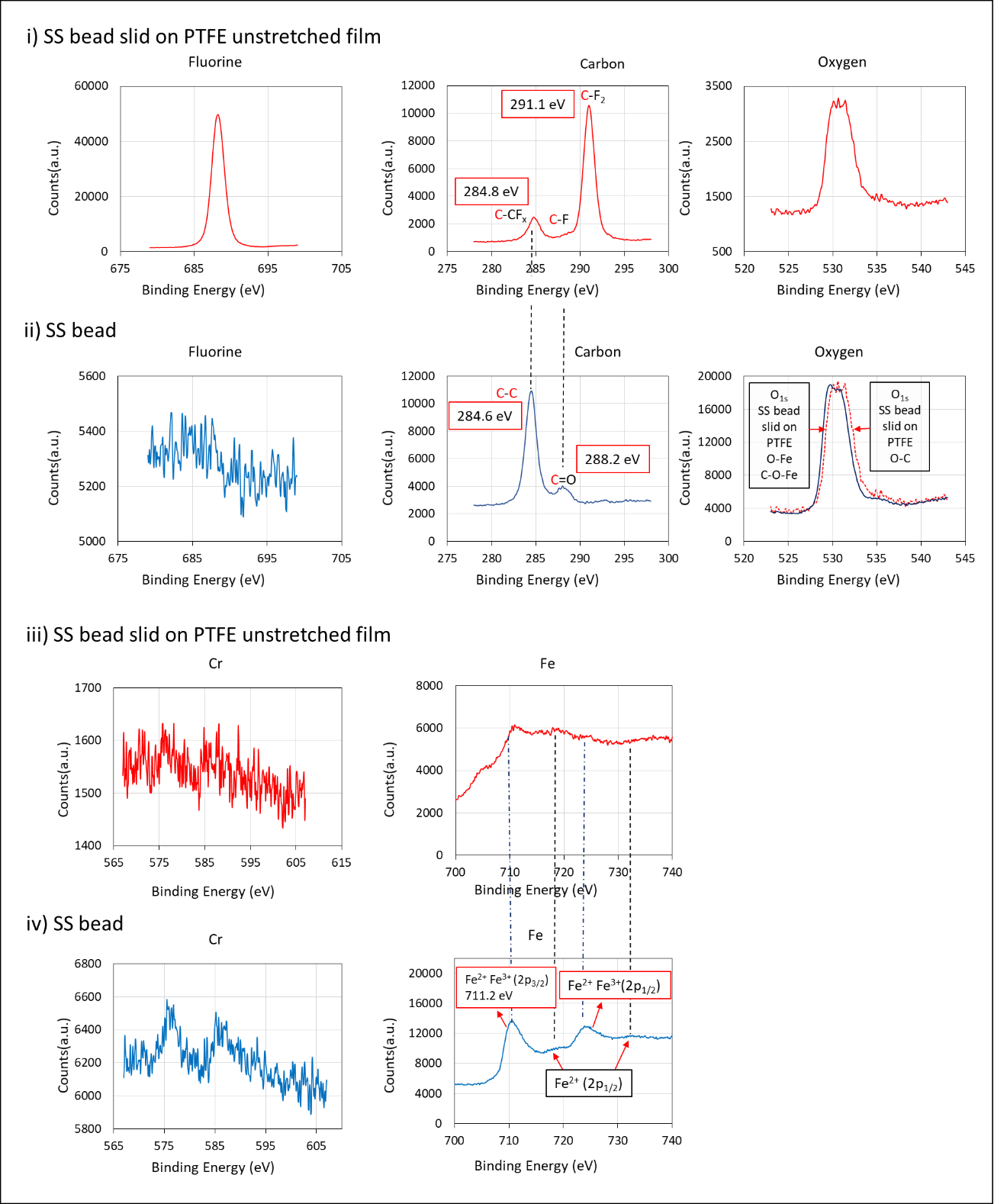
**

**Figure S8.** XPS elemental analysis ^5a,b^ of: i) SS bead that is slid on the unstretched PTFE, and ii) SS bead**.** The SS beads were cleaned using oxygen plasma before the sliding experiment and XPS analysis. The presence of the F_1s_ signal by sliding is evident for the PTFE transfer onto the. O_1s_ signal shifts to higher energies for the PTFE film transferred onto the SS metal bead by sliding.


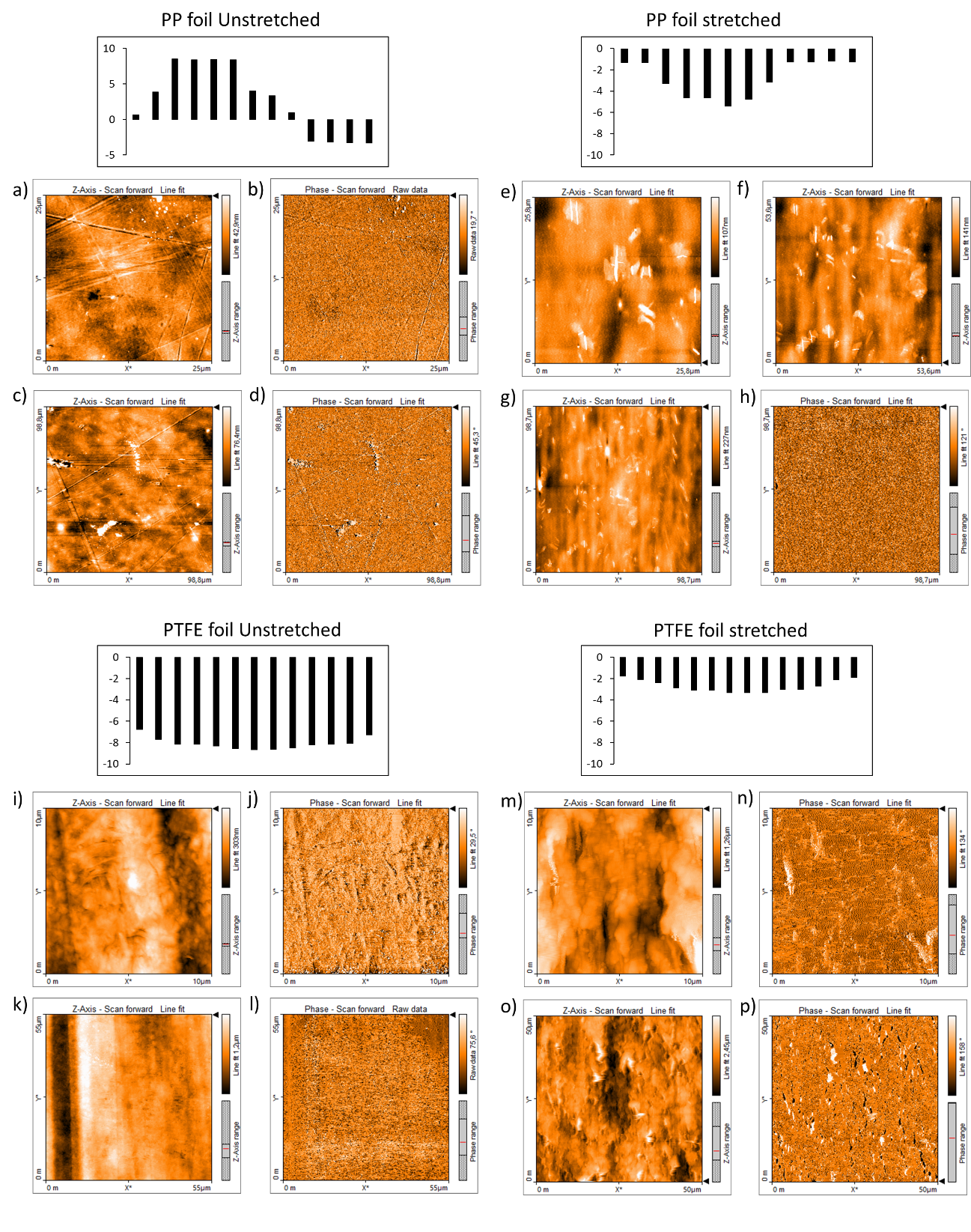


**Figure S9.** AFM height and phase images of unstretched and stretched PP (a -h) and PTFE (i - p). Surface potentials of PP and PTFE surfaces obtained from different samples are given in histograms above the KPFM surface potential images. Averaged surface potentials were used in the surface potentials given in the distribution plots. RH=30%, T= 25^o^C.

**
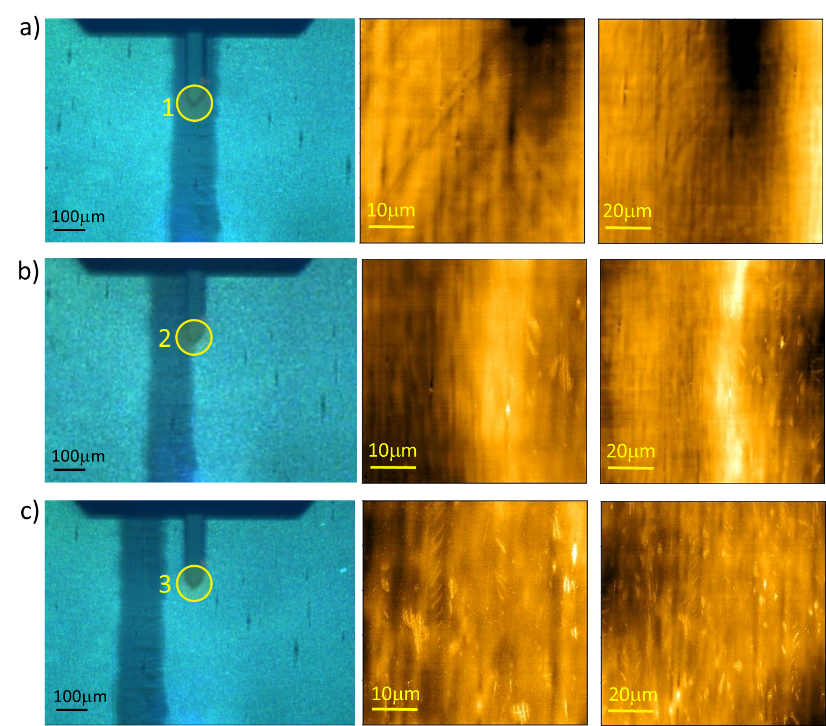
**

**Figure S10.** Left column: optical camera images of the AFM cantilever and the track of the SS bead (dark areas) formed on the stretched PP surface. Middle and right column: AFM height images at different regions on PP after SS bead was slid on the polymer. The images are obtained from a) the area where the SS bead was slid, b) the area that covers both the slid and unslid areas, and c) the area that belongs to a non-slid region.


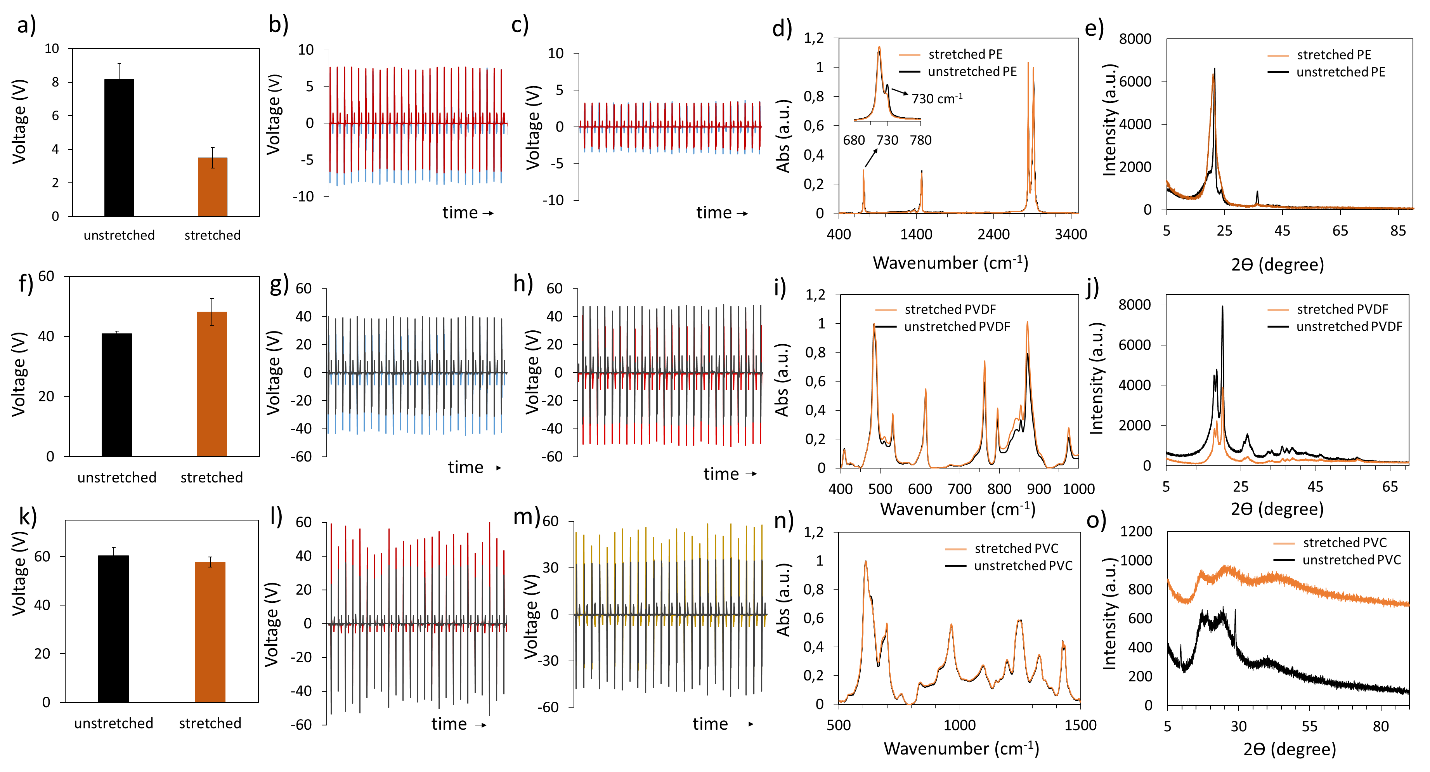


**Figure S11.** Contact-separation tribocharging, ATR-FTIR spectra, and XRD diffractograms of polymers before and after mechanically stretching: a) to e) poly(ethylene) (PE), f) to j) pol(vinylidenefloride) (PVDF), and k) to o) poly(vinylchloride) (PVC). Error bars given in (a), (f), and (k) correspond to standard deviations determined from at least five independent experiments. RH: 25-30%, T= 25^o^C.


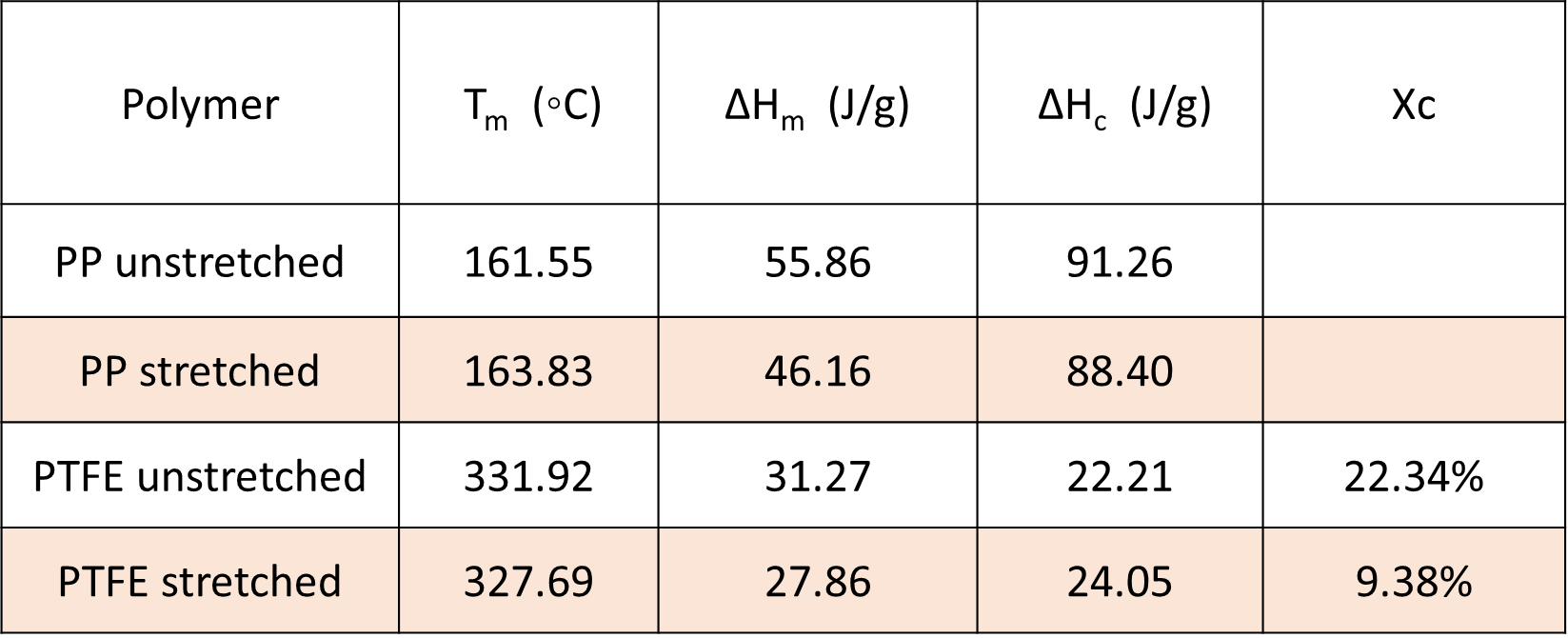


**Table S1.** DSC analysis (melting temperature, melting and crystallization enthalpy) of PP and PTFE before and after stretching.

| Polymer | HIT (MPa) | EIT (GPa) |
| --- | --- | --- |
| PP unstretched | 29.4 | 0.326 |
| PP stretched | 2304.7 | 195.3 |
| PTFE unstretched | 47.86 | 0.742 |
| PTFE stretched | 21.04 | 0.260 |

**Table S2.** Nanoindentation analysis: hardness (HIT) and elastic modulus (EIT) of PP and PTFE before and after stretching.


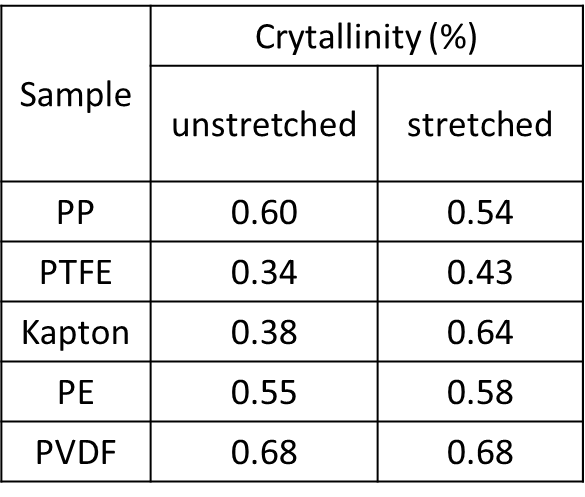


**Table S3.** Degree of crystallinity of some common polymers used in the current study before and after stretching. The degree of crystallinity increases in polymers (PTFE, Kapton and PE) after stretching except PP and PVDF. The ratio of the area of the crystalline peak(s) and the total area of the peak is used to calculate the crystallinity of the PTFE samples.

**Movie S1**: Triboelectric effect between SS metal rod and unstretched/stretched PP pieces.

**Movie S2**: Triboelectric effect between charged PTFE rod and unstretched/stretched PP pieces.

**Movie S3**: Triboelectric effect between charged skin and unstretched/stretched PP pieces.

**Movie S4**: Contact-separation mode triboelectric charging of unstretched polypropylene (PP).

**Movie S5**: Sliding mode triboelectric charging of unstretched polypropylene (PP) using a homemade sliding stage.

**References:**

1. A comparative study of characteristics of polytetrafluoroethylene fibers manufactured by various processes. Pretsch, E.; Bühlmann, P.; Badertscher, M. IR Spectroscopy. In Structure Determination of Organic Compounds: Tables of Spectral Data, Springer Berlin Heidelberg: Berlin, Heidelberg, pp 1-67, (2009).

2. a) Fazullin, D. D., Vitalevich, M. G., Sokolov, M. P., G. Shaikhiev, I. G., Infrared Spectroscopic Studies of the PTFE and Nylon Membranes Modified Polyaniline, Modern Applied Science 9(1), 242-249, (2015). b) IR spectroscopic studies of polytetrafluoroethylene and its modified forms. Russian Chemical Journal 3, 139-146). c) Oonuki, T., Araki, T., Oka, T., Matsuda, H., Shioya, N., Kano, J., Hibara, A. Hasegawa, T., Molecular Disaggregation process of PTFE using sodium chloride: a study by infrared spectroscopy, J.Phys.Chem.B., 129,4249−4255, (2025).

3. Self-lubrication mechanisms in polymer composites, A. I. Sviridyonok, Tribology International, 31-43, (1991).

4. a) Molecular-Level Reinforced Adhesion Between Rubber and PTFE Film Treated by Atmospheric Plasma Polymerization, Okubo, M., Onji, T., Kuroki, T., Nakano, H., Yao, E., Tahara, M., Plasma Chem Plasma Process 36, 1431–1448, (2016). b) Hallam, P. M., G-Mingot, M., Kampouris, D. K., Banks, C. E., Facile synthetic fabrication of iron oxide particles and novel hydrogen superoxide supercapacitors, RSC Adv., 2, 6672–6679, (2012).
